# Genomic insights into strategies used by *Xanthomonas albilineans* with its reduced artillery to spread within sugarcane xylem vessels

**DOI:** 10.1186/1471-2164-13-658

**Published:** 2012-11-21

**Authors:** Isabelle Pieretti, Monique Royer, Valérie Barbe, Sébastien Carrere, Ralf Koebnik, Arnaud Couloux, Armelle Darrasse, Jérôme Gouzy, Marie-Agnès Jacques, Emmanuelle Lauber, Charles Manceau, Sophie Mangenot, Stéphane Poussier, Béatrice Segurens, Boris Szurek, Valérie Verdier, Matthieu Arlat, Dean W Gabriel, Philippe Rott, Stéphane Cociancich

**Affiliations:** 1 CIRAD, UMR BGPI, F-34398 Montpellier Cedex 5, France; 2 CEA/DSV/IG/Génoscope, Centre National de Séquençage, F-91057 Evry Cedex France; 3 INRA, UMR LIPM, F-31326 Castanet-Tolosan Cedex France; 4 IRD, UMR RPB, F-34394 Montpellier Cedex 5, France; 5 INRA, UMR IRHS, F-49071 Beaucouzé France; 6 CNRS, UMR LIPM, F-31326 Castanet-Tolosan Cedex France; 7 Université de la Réunion, UMR PVBMT, F-97715 Saint-Denis La Réunion, France; 8 Université Paul Sabatier, UMR LIPM, F-31326 Castanet-Tolosan Cedex France; 9 University of Florida, Plant Pathology Department, Gainesville FL 32605 USA; 10 UMR BGPI, Campus International de Baillarguet, TA A-54/K, F-34398 Montpellier Cedex 5, France

## Abstract

**Background:**

*Xanthomonas albilineans* causes leaf scald, a lethal disease of sugarcane. *X. albilineans* exhibits distinctive pathogenic mechanisms, ecology and taxonomy compared to other species of *Xanthomonas*. For example, this species produces a potent DNA gyrase inhibitor called albicidin that is largely responsible for inducing disease symptoms; its habitat is limited to xylem; and the species exhibits large variability. A first manuscript on the complete genome sequence of the highly pathogenic *X. albilineans* strain GPE PC73 focused exclusively on distinctive genomic features shared with *Xylella fastidiosa*—another xylem-limited *Xanthomonadaceae*. The present manuscript on the same genome sequence aims to describe all other pathogenicity-related genomic features of *X. albilineans*, and to compare, using suppression subtractive hybridization (SSH), genomic features of two strains differing in pathogenicity.

**Results:**

Comparative genomic analyses showed that most of the known pathogenicity factors from other *Xanthomonas* species are conserved in *X. albilineans*, with the notable absence of two major determinants of the “artillery” of other plant pathogenic species of *Xanthomonas*: the xanthan gum biosynthesis gene cluster, and the type III secretion system Hrp (hypersensitive response and pathogenicity). Genomic features specific to *X. albilineans* that may contribute to specific adaptation of this pathogen to sugarcane xylem vessels were also revealed. SSH experiments led to the identification of 20 genes common to three highly pathogenic strains but missing in a less pathogenic strain. These 20 genes, which include four ABC transporter genes, a methyl-accepting chemotaxis protein gene and an oxidoreductase gene, could play a key role in pathogenicity. With the exception of hypothetical proteins revealed by our comparative genomic analyses and SSH experiments, no genes potentially involved in any offensive or counter-defensive mechanism specific to *X. albilineans* were identified, supposing that *X. albilineans* has a reduced artillery compared to other pathogenic *Xanthomonas* species. Particular attention has therefore been given to genomic features specific to *X. albilineans* making it more capable of evading sugarcane surveillance systems or resisting sugarcane defense systems.

**Conclusions:**

This study confirms that *X. albilineans* is a highly distinctive species within the genus *Xanthomonas*, and opens new perpectives towards a greater understanding of the pathogenicity of this destructive sugarcane pathogen.

## Background

Members of the *Xanthomonas* genus are Gram-negative bacteria belonging to the γ-subdivision of the *Proteobacteriae* and to the *Xanthomonadaceae* family. They are exclusively plant-associated bacteria. The plant pathogenic xanthomonads target over 120 monocotyledonous species (including rice, sugarcane, banana, etc.) and over 260 dicotyledonous species (e.g. citrus, cauliflower, cabbage, bean, pepper, etc.), causing dramatic economic losses worldwide. A recently published comprehensive list of plant pathogenic bacteria divided the members of the *Xanthomonas* genus into 27 species and, at the infra-species level, into over 120 pathovars [1]. Over the past few decades, many attempts have been made to classify members of the *Xanthomonas* genus using a large variety of biochemical, pathogenicity-based or genomic methods [2-5]. A recent extensive multilocus sequence analysis (MLSA) performed with 119 strains spanning the whole *Xanthomonas* genus clearly showed that the strains are distributed into two uneven groups, with group 2 containing all but five species, namely *X. albilineans*, *X. sacchari*, *X. theicola*, *X. hyacinthi* and *X. translucens*, which were clustered into group 1 [5]. This distribution is consistent both with that based on 16S rDNA sequences [6] and with a distribution based on intergenic sequences between 16S rDNA and 23S rDNA [7]. To date, four strains belonging to group 1 were sequenced, *i.e. X. albilineans* strain GPE PC73 [8] and *Xanthomonas* spp. strains NCPPB4393, NCPPB1131 and NCPPB1132 [9]. *Xanthomonas spp.* strain NCPPB4393 was isolated from an insect collected on a diseased banana plant, but ended by being described as *X. sacchari* which was also isolated on sugarcane and milled rice [10,11]. However, no disease caused by this species on any plants has been described to date. *Xanthomonas* spp. strains NCPPB1131 and NCPPB1132 were both isolated from *Musa* and belong to unidentified species, although they are phylogenetically related to *X. albilineans* and to *X. sacchari*, respectively [9].

*Xanthomonas albilineans* is the causal agent of leaf scald—one of the major diseases of sugarcane (*Saccharum* spp.) that occurs in at least 66 countries worldwide [12]. Host plants for this species are restricted to some *Poaceae*, of which sugarcane is the main known target. Disease symptoms vary from a single, white, narrow, sharply defined stripe to complete wilting and necrosis of infected sugarcane leaves, leading to plant death and large yield losses in the field. *X. albilineans* produces albicidin, a secreted antibiotic with phytotoxic properties [13]. Albicidin is a potent DNA gyrase inhibitor that targets chloroplastic DNA gyrase A, inhibits chloroplast DNA replication and blocks chloroplast differentiation, resulting in the white foliar stripe symptom [13,14]. Dissemination of *X. albilineans* occurs mainly mechanically through use of contaminated harvesting tools and by distribution and planting of infected cuttings. However, aerial transmission and potential for epiphytic survival have also been reported for this pathogen [15-17]. The sugarcane leaf scald outbreaks that occurred in the late 1980s and early 1990s, especially in Guadeloupe [18], Cuba [19], Florida [20], Taiwan [21], Louisiana [22] and Texas [23], were associated with strains of *X. albilineans* belonging to a specific genetic sub-group known as pulsed-field gel electrophoresis group B (PFGE-B) [24]. The complete genome sequence of strain GPE PC73 (= CFBP 7063) of *X. albilineans* from Guadeloupe, which belongs to PFGE-B, was published recently (NCBI reference sequence: NC_013722.1). Previous pathogenicity studies identified this strain as highly pathogenic in sugarcane [25]. The genetic information of *X. albilineans* is encoded on a 3.8-Mb circular chromosome, much smaller than those described to date in other sequenced *Xanthomonas* (approximately 5 Mb), and on three plasmids of 25, 27 and 32 kb, respectively. The G+C content is 63%, close to values from other sequenced *Xanthomonas* genomes, and 3115 protein-coding sequences have been annotated manually [8].

Within its sugarcane host, the habitat of *X. albilineans* is limited to xylem [13,26]. Our earlier study of the genome sequence of *X. albilineans* strain GPE PC73 [8] focused on the description of genomic features shared with an evolutionary distant *Xanthomonadaceae* that is also xylem-limited: *Xylella fastidiosa*—the causal agent of numerous lethal plant diseases [27]. This first paper, however, did not describe pathogenicity-related features shared by *X. albilineans* and other species of *Xanthomonas*. This initial study by Pieretti et al. showed that the two xylem-limited *Xanthomonadaceae* experienced convergent reductive genome evolution during their descent from a common ancestral parent and possess similar cellulases, which are adapted to the use of plant cell breakdown products as carbon source. These genomic features are not shared with any of the other sequenced species of *Xanthomonas*. Another genomic feature common to *X. albilineans* and *X. fastidiosa* is the absence of a type III secretion system (T3SS) of the Hrp1 and Hrp2 (hypersensitive response and pathogenicity-1 and 2, respectively) injectisome families that are used by most Gram negative phytopathogenic bacteria to deliver bacterial effector proteins or virulence factors into the host plant cell. This latter genomic feature is shared with other sequenced species of group 1 (*X. sacchari* and the two *Xanthomonas* spp. isolated on *Musa*), but not with sequenced species of group 2. The genomic features shared between *X. albilineans* and *X. fastidiosa* were proposed to be associated with the cloistered environmental niche of xylem vessels and to adaptation to nutrient-poor xylem elements [8]. However, *X. albilineans* and *X. fastidiosa* also differ in several important genomic features: (i) genome erosion was less important in *X. albilineans* than in *X. fastidiosa* (890 genes lost by *X. fastidiosa* were conserved in *X. albilineans*); (ii) *X. albilineans* lost xanthan gum biosynthesis genes that were partially conserved by *X. fastidiosa*; and (iii) only 11 genes are specific to the two xylem-limited *Xanthomonadaceae*[8]. *X. albilineans*, while sharing important genomic features with *X. fastidiosa*, remains closer to xanthomonads than to this distant cousin species from another genus.

The only pathogenicity factor of *X. albilineans* that has been studied extensively to date is albicidin. Some albicidin-deficient mutants are unable to produce disease symptoms, indicating that this toxin potentially plays an important role in disease expression [13]. However, other albicidin-deficient mutants are still able to produce severe leaf symptoms, indicating that albicidin alone cannot fully explain the pathogenicity mechanism(s) of *X. albilineans*[28]. Albicidin production *in vitro* also varies according to the strain of the pathogen [29]. However, no relationship was found between the amount of albicidin produced *in vitro* and the pathotypes or genetic diversity of the pathogen [29].

The present study of the genome sequence of *X. albilineans* strain GPE PC73 aimed to identify other virulence factors of *X. albilineans* in order to gain further insights into the pathogenicity of this sugarcane pathogen. To this end, we listed and described all predicted pathogenicity-related genomic features and compared, by suppression subtractive hybridization (SSH), two strains of *X. albilineans* that differ in pathogenicity. The genome sequence of *X. albilineans* strain GPE PC73 was compared to eight available genome sequences of *XanthomonasX. axonopodis* pv. *vesicatoria* strain 85–10 [30], *X. axonopodis* pv. *citri* strain 306 [31], *X. campestris* pv. *campestris* strain ATCC 33913 [31], *X. campestris* pv*. vasculorum* NCPPB702 [32], *X. campestris* pv. *musacearum* NCPPB4381 [32], *X. oryzae* pv. *oryzae* MAFF 311018 [33], *X. oryzae* pv. *oryzicola* strain BLS256 [34] and *X. sacchari*[9]]. Genomic features shared between these eight genomes and *X. albilineans* strain GPE PC73 were surveyed in order to better understand the strategies developed by *X. albilineans* to compensate for the absence of the xanthan gum biosynthesis genes and the type III secretion system Hrp. In the rather cloistered environmental niche inside xylem vessels, *X. albilineans* may have largely avoided surveillance by general and specific plant defense systems. The absence of T3SS Hrp may be partially palliated due to the xylem-limited lifestyle of *X. albilineans*, as this pathogen lives and multiplies essentially in a dead-cell environment. However, like other bacterial vascular pathogens, *X. albilineans* may interact with living xylem parenchyma cells through pit membranes [35]. Genomic features of *X. albilineans* strain GPE PC73 were also analyzed to better understand the adaptation of *X. albilineans* to the nutrient-poor xylem elements within xylem vessels.

## Results and discussion

### Secretion systems

To date, six different types of secretion systems in Gram-negative bacteria have been described, differing both in their structures and in the molecules transferred, which can be proteins, including both peptides and enzymes, and other small and large molecules, including DNA [36]. The secreted molecules can play an important role in cellular homeostasis and even in bacterial lifestyles: some are literally injected into host cells in which they modify host physiology to promote colonization.

In order to establish themselves successfully in their hosts, *Xanthomonas* species rely on the presence of various types of secretion systems [37,38]. The type I secretion system (T1SS) is required for secretion of a variety of degradative enzymes and offensive molecules, including antibiotics and molecules involved in plant or animal pathogenicity. These include a variety of hydrolases (proteases, phosphatases, esterases, nucleases and glucanases) and proteic toxins (hemolysins or bacteriocins) [39,40]. T1SS utilises an outer membrane protein factor, TolC, which traverses both the periplasm and the outer membrane [40-43]. TolC is also essential for the action of multidrug resistance (MDR) efflux pumps, and was shown to be required for pathogenicity of *X. fastidiosa*[44] and for secretion of an elicitor in *X. oryzae* pv*. oryzae*[45]. The gene encoding TolC in *X. albilineans* (XALc_0747) shares 32% amino acid identity with the *tolC* gene of *Escherichia coli* strain K12, and more than 61% amino acid identity with the TolC protein of *X. fastidiosa* or other xanthomonads. Like other xanthomonads, and unlike *X. fastidiosa*, *X. albilineans* carries other outer membrane factor genes sharing some similarities with *tolC*. These are located in the same genomic regions as genes encoding MDR efflux pumps. They share less than 20% amino acid identity with the *tolC* gene of *E. coli.* Six of these genes are conserved in other *Xanthomonas* strains (XALc_0344, XALc_1555, XALc_2009, XALc_2167, XALc_2468 and XALc_3155) whereas a seventh is specific to *X. albilineans* (XALc_1770).

The type II secretion system (T2SS) secretes cell wall-degrading enzymes like cellulases and xylanases. Two independent T2SS gene clusters, Xps and Xcs, are apparently dedicated to distinct substrates and roles in the genus *Xanthomonas*[31,46]. Both are present in *X. axonopodis* pv. *vesicatoria*, *X. campestris* pv. *campestris*, *X. axonopodis* pv. *citri*, *X. fuscans* subsp. *aurantifoli*, *X. gardneri* and *X. perforans.* Xcs is missing in the *X. oryzae* pv. *oryzae* and *X. oryzae* pv. *oryzicola* genomes, whereas only two genes of the cluster (*xcsN* and *xcsM*, respectively) are conserved in *X. campestris* pv. *vasculorum* and *X. campestris* pv. *musacearum*. The chromosome of *X. albilineans* strain GPE PC73, like the chromosome of *X. sacchari,* possesses only the *xps* gene cluster, which contains 11 genes annotated as *xpsD* to *xpsN* (XALc_2654 to XALc_2664). The Xps T2SS of *X. axonopodis* pv. *vesicatoria* has been shown to be involved in pathogenicity [46]. The contribution to virulence of *X. albilineans* of the Xps T2SS is so far unknown, but 364 genes are found in the genome that encode proteins predicted to be T2SS-secreted using SignalP 4.0 server http://www.cbs.dtu.dk/services/SignalP/ (see Additional file 1). They include 159 hypothetical proteins as well as many additional proteins known to be secreted by a T2SS.

**Figure 1:**
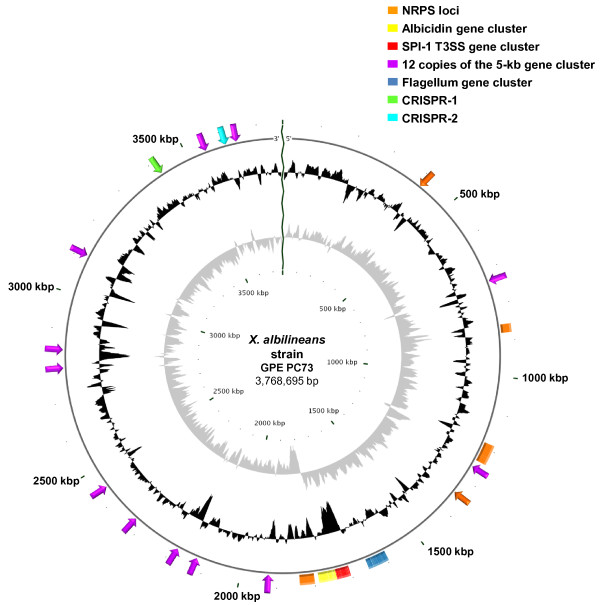
**Circular representation of the chromosome of strain GPE PC73 of *X. albilineans*.** The scale is shown in kbp in the center and around the periphery. Boxes: Albicidin biosynthesis gene cluster (yellow), other large NRPS loci (orange), T3SS SPI-1 gene cluster (red) and flagellum gene cluster (blue). Arrows: 12 copies of the 5-kb gene cluster present at 12 different locations of the chromosome (purple), locus CRISPR-1 (green), locus CRISPR-2 (sky blue) and short NRPS loci containing only a short NRPS gene (orange). The grey circle shows the GC skew (G-C)/(G+C) using a 100-base window. The black circle shows the G+C content using a 100-base window

Type III secretion systems (T3SS), also called injectisomes, are molecular syringes that deliver into the cytoplasm of host cells effector proteins capable of targeting and disrupting key functions of the host. Seven T3SS families have been described [47], but phytopathogenic bacteria usually rely on T3SS from the Hrp families to fulfil their pathogenicity. The remarkable absence of a T3SS-Hrp in the genome of *X. albilineans* implies that secretion of effectors interacting with sugarcane cells relies on other secretion systems. Interestingly, a T3SS gene cluster belonging to the SPI-1 (for *Salmonella* Pathogenicity Island-1) injectisome family, found mainly in animal pathogens and insect symbionts, is present in the genome of *X. albilineans* strain GPE PC73 (XALc_1472 to XALc_1511) near the termination site of chromosome replication (Figure 1). Genomic and evolutionary features of the T3SS SPI-1 of *X. albilineans* were described recently [48]. This system, which is partially conserved in *X. axonopodis* pv. *phaseoli*, shares only low similarity with other available T3SS SPI-1 sequences but encodes all components required for secretion [48]. However, functional analysis with knockout insertional mutants showed that the T3SS SPI-1 is not required by *X. albilineans* to spread within the xylem and to cause disease. Involvement of a T3SS SPI-1 in adherence to plant surfaces was proposed for the strictly epiphytic *Erwinia tasmaniensis* strain Et1/99 [49] suggesting that the T3SS SPI-1 of *X. albilineans* may be involved in leaf surface colonization. Alternatively, T3SS SPI-1 may play an important role in association with an animal host, although insect vectors of *X. albilineans* have not yet been reported. 

**Figure 2:**
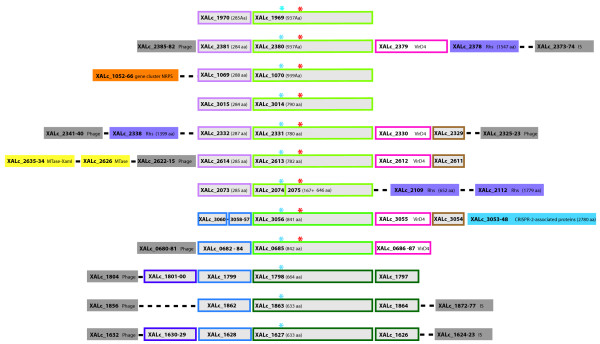
**Representation of the 12 copies of the 5-kb gene cluster present at 12 different locations within the chromosome of strain GPE PC73 of *X. albilineans.*** The light-coloured boxes correspond to the ORFs of the 5-kb gene cluster. They are bordered by unframed plain-coloured boxes assigned to phage, IS transposases, virD4, Rhs or other specific sequences. Similar ORFs (orthologs) are represented by the same coloured squares. The dotted line illustrates two non-adjacent ORFs. The blue and the red stars above the green framed boxes show positions of the serine from the triade Ser-His-Asp and of the Poly-S locations, respectively. MTase: DNA methyltransferase. MTase-XamI: XamI DNA methyltransferase-restriction endonuclease system

The type IV secretion system (T4SS) has been described as an important bacterial factor helping bacterial adaptation to new hosts [50]. A diversity of structures and functions has been reported as well as the versatile nature of this system, which can be used to mediate horizontal gene transfer or to secrete virulence factors [51]. In *Agrobacterium* and *Helicobacter*, VirB2 and VirB5 form an extracellular pilus that was proposed to be an adhesion-like protein susceptible to interact with specific host-cell receptors [52]. A recent study demonstrated that the T4SS of *Bartonella* mediates host-specific adhesion to erythrocytes [53]. The chromosome of *X. albilineans* strain GPE PC73 possesses a T4SS, which may correspond to an ancestral gene cluster because genes encoding this system share 53–83% amino acid identity with genes located at the same position in other xanthomonads, *i.e.* downstream of the same *uvrB* gene (XALc_1870) and the same valine tRNA (XALc_1869). However, this T4SS seems to be complete only in *X. axonopodis* pv. *citri*, in which the protein encoded by XAC2622 was characterized structurally recently by NMR and X-ray crystallography as an outer membrane transport protein, confirming that this protein corresponds to VirB7 [54]. The T4SS gene cluster of *X. albilineans* strain GPE PC73 possesses the same *virB7* gene (XALc_1843) and contains a phage-related sequence (XALc_1844 to XALc_1866) but lacks both the *virB5* gene and the *virD4* gene that encodes an NTPase required to power the conjugation/secretion apparatus. Nonetheless, four putative *virD4*-like genes (XALc_2330, XALc_2379, XALc_2612 and XALc_3055) are present on the chromosome in a repeated region (see below and Figure 2) and an additional putative *virD4*-like gene (XALr_3251) is present on one of the three plasmids, suggesting that trans-complementation of this gene might occur. Still, no functional T4SS involving a VirD4 encoded elsewhere in the genome (*i.e.* not encoded in the T4SS gene cluster) has been described in any bacterium to date. The missing *virB5* gene may also be complemented by *virB5*-like genes present elsewhere in the genome. Each of the three plasmids present in *X. albilineans* strain GPE PC73 harbors an incomplete conjugal transfer system similar to a T4SS, which includes a VirB5-like protein (XALp_3186, XALq_3225 and XALr_3258). Another putative *virB5-*like gene (XALc_2643) is present in a phage-related region of the chromosome. 

Type V secretion systems (T5SS) form a heterogeneous family of transporters for non-fimbrial adhesins. Three subtypes have been described for this family: autotransporter, trimeric autotransporter and two-partner system (TPS) [37,55,56]. Since we have identified genes encoding non-fimbrial adhesins in the genome of *X. albilineans* GPE PC73 (see below), one might expect the occurrence of at least one T5SS.

In contrast to most of the xanthomonad species, *X. albilineans* strain GPE PC73 does not possess a type VI secretion system (T6SS)*.* The genome of *X. oryzae* pv. *oryzae* strain MAFF 311018 contains two T6SS gene clusters, while the genome of *X. axonopodis* pv. *vesicatoria* strain 85–10 harbours only one copy split over two loci [57]. Despite T6SSs being found in pathogenic and nonpathogenic bacteria, this system has been shown to be essential for pathogenicity in many animal pathogens [58,59].

In *X. albilineans*, four secretion systems are potentially involved in secretion of pathogenicity effectors, namely T1SS, T2SS, T5SS and T3SS SPI-1. This latter system may be involved in interactions with an animal host rather than with sugarcane. In *X. oryzae* pv. *oryzae,* the elicitor Ax21, formerly known as AvrXa21, has been described as being secreted by a T1SS [60,61]. As with Ax21, further functional analysis and biochemical experimentation will be necessary to identify such effectors in *X. albilineans*. Small molecules, which are secreted by specific transporters usually encoded within their biosynthesis gene clusters, are also good candidates for secretion into the xylem by *X. albilineans*. These could then diffuse into targeted adjacent living parenchymal cells and act as pathogenicity effectors, or may enter plant cells by hijacking nutrient transporters.

### Nonribosomal peptide synthetase genes

Bacteria use nonribosomal peptide synthetases (NRPSs) to produce peptides or small molecules of broad structural and biological activity that can contribute to virulence, adaptation to unfavorable environments or competition with rival microorganisms in their natural habitat (for reviews, [62,63]). Small molecules synthesized by NRPS may play an important role in the pathogenicity of *X. albilineans*. This assumption is based on the presence in the genome of *X. albilineans* strain GPE PC73 of 12 genes encoding NRPSs comprising 4% of the chromosome (Figure 1). Three of these NRPS genes belong to the albicidin biosynthesis gene cluster XALB1, which was sequenced previously from *X. albilineans* strain Xa23R1 [64]. Albicidin, which is secreted by *X. albilineans* using a specific transporter encoded by XALB1, can enter sugarcane chloroplasts using nucleoside transporters similar to the nucleoside transporter Tsx, which mediates albicidin uptake in *E. coli*[65]. The structure and function of small molecules synthesized by the nine other NRPS genes identified in the genome of *X. albilineans* strain GPE PC73 are currently unknown. *In silico* features of these nine new NRPS genes were analyzed and seven of these genes are predicted to share features with NRPS genes identified in the genome of other *Xanthomonas* spp. strains also associated with monocotyledonous species (*X. oryzae* pv. *oryzae, X. oryzae* pv. *orizycola* and *X. translucens*) (Royer et al., unpublished data). This suggests that unknown small molecules synthesized by these NRPS gene clusters are involved in specific interaction with these plants (Royer et al., unpublished data). Furthermore, four of these new NRPS genes are grouped in a gene cluster encoding a specific transporter, suggesting that at least one small molecule synthesized by this gene cluster is secreted and may interact with sugarcane cells. Small molecules synthesized by NRPS that act as surfactants have been described for *Pseudomonas syringae*, which is found in various environments and can survive on leaf surfaces as an epiphyte by producing several surfactants synthesized by NRPSs [66,67]. NRPSs have also been described as being involved in the biosynthesis of siderophores [68]. These iron-chelating structures are used widely by phytopathogenic bacteria to achieve survival in iron-depleted environments or to out-compete other bacterial strains in the same environment [69]. To date, NRPS genes are the only genes identified in the genome of *X. albilineans* strain GPE PC73 that can be considered as good candidates to produce the small molecules required for interactions with sugarcane cells.

### Cell wall degrading enzymes

The genome of *X. albilineans* strain GPE PC73 exhibits 19 genes encoding putative cell wall degrading enzymes (e.g. cellulases, polygalacturonases, rhamnogalacturonases, beta-glucosidases and xylanases), all of which being predicted to be secreted by the Xps-T2SS (see Additional file 1). As previously described, *X. albilineans* and *X. fastidiosa* possess similar enzymes that help adapt these organisms to use plant cell breakdown products as carbon sources [8]. Such enzymes include endoglucanase EngXCA and 1,4-beta cellobiosidase CbhA which, in both of the xylem-limited *Xanthomonadaceae*, but not in other *Xanthomonadaceae*, have a cellulose binding domain (CBD) at their C-terminal extremity, and a long linker region consisting of simple or repetitive sequences rich in proline, threonine, serine, or glycine. Such long linker regions are known to enhance substrate accessibility [70,71], indicating that these enzymes are particularly well adapted to the degradation of carbohydrate substrates.

**Figure 3:**
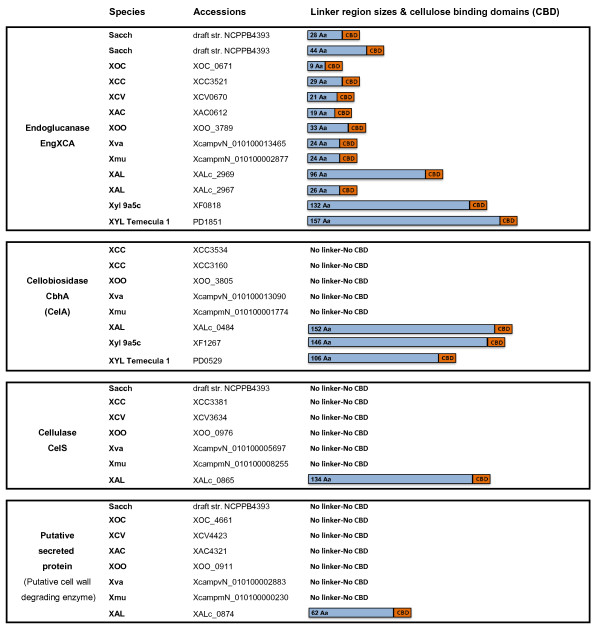
**Comparison of four cell wall degrading enzymes encoded by *Xanthomonadaceae *species.** Sacch: *X. sacchari* strain NCPPB4393; XOC: *X. oryzae* pv. *oryzicola* strain BLS256; XCC: *X. campestris* pv. *campestris* strain ATCC 33913; XCV: *X. campestris* pv. *vesicatoria* strain 85–10; XAC: *X. axonopodis* pv. *citri* strain 306; XOO: *X. oryzae* pv. *oryzae* strain MAFF 311018; Xva: *X. campestris* pv. *vasculorum* strain NCPPB702; Xmu: *X. campestris* pv*. musacearum* strain NCPPB4381; XAL: *X. albilineans* strain GPE PC73; Xyl 9a5c: *X. fastidiosa* strain 9a5c; XYL Temecula 1: *X. fastidiosa* strain Temecula1. Blue boxes: linker regions consisting of simple or repetitive sequences rich in proline, threonine, serine, or glycine; box length is proportional to the length of linker region (number of amino acids of each linker region is indicated inside each box). Orange boxes: cellulose binding domains (CBD). Aa = amino acids

*X. albilineans* possesses one copy of the 1,4-beta cellobiosidase *cbhA* gene (XALc_0484). Interestingly, the 1,4-beta cellobiosidase *cbhA* gene is missing in the non vascular *Xanthomonas* species (*X. oryzae* pv. *oryzicola*, *X. axonopodis* pv*. citri*, *X. axonopodis* pv. *vesicatoria* and other sequenced xanthomonads including *X. sacchari*) but is conserved in the xylem-invading *Xanthomonas* species (*X. oryzae* pv. *oryzae*, *X. campestris* pv*. campestris, X. campestris* pv*. vasculorum* and *X. campestris* pv. *musacearum*). However, in the latter species, CbhA has neither a linker region nor a CBD (Figure 3). The *cbhA* gene was shown to contribute to virulence of the xylem-invading pathogen *Ralstonia solanacearum*[72]*.* The presence of a *cbhA* gene in the xylemic xanthomonads *X. fastidiosa* and *R. solanacearum* suggests that this enzyme is absolutely required for spread within xylem vessels. A specific feature of these vessels is that they are interconnected by channels, called bordered pits, which allow the passage of xylem sap but block the passage of larger objects due to the presence of a pit membrane. This pit membrane constitutes the primary cell wall barrier that separates adjacent xylem water conduits, limiting in particular the colonization of the plant by a pathogen [73]. The movement of *X. fastidiosa* within the plant is an active process and appears to depend on its ability to disrupt pit membranes (for a review, see [27]). The pit membrane is composed of pectin and cellulose microfibrils [74], and may require several enzymes for complete dissolution. The presence of a *cbhA* gene in the xylemic xanthomonads, *X. fastidiosa* and *R. solanacearum*, might indicate that this enzyme is required for degradation of the pit membrane. *X. albilineans* possesses two polygalacturonase genes (XALc_0811 and XALc_1916) that are conserved in all sequenced *Xanthomonas* and in *R. solanacearum* and potentially required for degradation of pectin. XALc_0811 is not conserved in *X. fastidiosa*. XALc_1916 is not conserved in strain 9a5c of *X. fastidiosa* (isolated from citrus), but is conserved in strain Temecula1 of *X. fastidiosa* (isolated from grapevine) where it was shown to be required for colonization and pathogenicity of this strain in grapevine [75]. 

The genome of *X. albilineans* strain GPE PC73 contains two copies of the endoglucanase *engXCA* gene (XALc_2969 and XALc_2967). Endoglucanase EngXCA is conserved in all other *Xanthomonas* and also possesses a linker region and a CBD, but the linker region in these species is much smaller than that in *X. albilineans* and *X. fastidiosa* (Figure 2). Interestingly, the genome of *X. albilineans* strain GPE PC73 encodes two additional enzymes harboring a long linker region and a CBD at their C-termini, namely cellulase CelS (XALc_0865) and a putative secreted protein (XALc_0874), which may be considered as putative cell wall degrading enzymes because of the presence of a CBD. The cellulase gene *celS* has a frameshift mutation in *X. axonopodis* pv. *citri*, is not present in *X. fastidiosa* and is conserved in seven other sequenced genomes of *Xanthomonas,* but it does not possess any linker region or any CBD in these species. The putative cell wall degrading enzyme XALc_0874 is absent in both *X. campestris* pv. *campestris* and *X. fastidiosa.* It is, however, present in the seven other sequenced genomes of *Xanthomonas,* but does not possess any linker region or any CBD in these species (Figure 3). The presence of five genes that encode enzymes harboring a long linker region and a CBD at their C-termini indicates that *X. albilineans* is adapted to the utilization of cell breakdown products as a carbon source. Xylem is a water transport network of vessels composed of dead, lignified cells. Xylem sap is therefore rich in cell-wall breakdown products. *X. albilineans* strain GPE PC73 encodes putative sugar transporter systems, further supporting the concept that cell-wall derived sugars are consumed by this pathogen.

### TonB-dependent transporters

Outer membrane TonB-dependent transporters (TBDT) are involved in the active transport of plant nutrients, more precisely iron-siderophore complexes, vitamin B12, nickel or other large molecules such as plant carbohydrates [76,77]. A large proportion of TBDT genes were described as related to carbohydrate utilization in *X. campestris* pv. *campestris*[76]. There is a large variation in the number of TBDT genes among xanthomonads, even at the infra-species level. This number does not seem to be linked to genome size but rather to the ecological niche and lifestyle of the species considered [77]. Seventy-two TBDT genes were identified in the genome of *X. campestris* pv. *campestris*, of which only nine were assigned to iron uptake; several others were associated with plant carbohydrate utilization and the pathogenicity of this species [76]. Thirty-five putative TBDT genes were found in the chromosome of *X. albilineans* strain GPE PC73, including 26 canonical TBDTs and five TBDT/Oar-like genes. Orthologous or paralogous genes were found in other sequenced genomes of *Xanthomonas*, except for gene XALc_1949 which encodes a non-canonical TBDT/Oar-like protein specific to *X. albilineans* and for gene XALc_2962 which encodes a canonical TBDT specific to *X. albilineans* and *X. sacchari*. The presence of 35 TBDT genes in *X. albilineans* indicates that this species, like other species of *Xanthomonas*, is adapted to plant scavenging and to living in nutrient-poor environments. A recent transposon mutagenesis study of strain XaFL07-1 of *X. albilineans* revealed the presence of two pathogenicity-related TBDT loci (XALc_0643 and XALc_0723) involved in disease severity and extent of stalk colonization, although orthologous genes of these two TBDTs have not yet been reported as pathogenicity factors in other xanthomonads [28]. Further studies will be necessary to characterize the nature of the nutrients transported by these two TBDTs. XALc_0643 is present in a putative operon upstream from another TBDT (XALc_0646) and a putative nucleoside hydrolase. XALc_0723 is present just downstream of a putative HpcH/HpaI aldolase involved in the (d)-glucarate/galactarate catabolic pathway. These three TBDTs (XALc_0643, XALc_0646 and XALc_0723) may be adapted to the transport of carbohydrate products provided by the five cellulases specific to *X. albilineans*.

### Lipopolysaccharides

Lipopolysaccharides (LPS) are essential for protecting the cell from hostile environments. They can also play a direct role in interactions between bacteria and eukaryotic host cells. In animal pathogenic bacteria, *lps* loci involved in LPS biosynthesis are under host selection, with large variations in the *lps* gene cluster being found [78]. Similarly, the *lps* loci of plant pathogenic bacteria are also under selection, especially to escape host defense responses. Bacterial LPSs have been found to act as elicitors of plant innate immunity. In *Xanthomonas axonopodis* pv. *citri*, the O-antigen moiety of the LPS has been shown recently to act as a PAMP (Pathogen-Associated Molecular Pattern) and therefore to activate the basal response of the attacked plant, in particular by inducing the expression of defense-related genes and promoting callose deposition, the latter being accompanied by an oxidative burst [79]. Comparison of the LPS biosynthetic gene clusters of *Xanthomonas* species shows a high variability in the number and identity of genes [80,81]. Multiple horizontal gene transfer events are considered to be responsible for the high variation in LPS biosynthetic gene clusters between various xanthomonads [82]. LPS clusters are bordered by the highly conserved *etfA* and *metB* genes in all xanthomonads sequenced to date. In *X. albilineans* strain GPE PC73, the LPS gene cluster is also bordered by *etfA* (XALc_2699) and *metB* (XALc_2712), and comprises 12 genes (XALc_2700 to XALc_2711), five of which are specific to *X. albilineans* (XALc_2700 to XALc_2704). Seven genes (XALc_2705, XALc_2706 (*gmd*), XALc_2707 (*rmd*), XALc_2708, XALc_2709, XALc_2710 (*xzm*) and XALc_2711) show the highest similarity with genes from the vascular sugarcane pathogen *X. campestris* pv. *vasculorum* (see Additional file 2). The relatedness of LPS-encoding genes between *X. albilineans* strain GPE PC73 and *X. campestris* pv. *vasculorum* is incongruent with their phylogenetic relationship, which separates the two pathogens into two distinct and distant clades [5]. In the draft genome sequence of *X. campestris* pv. *vasculorum*, the LPS cluster is found at the borders of two contigs (Additional file 2). One contig contains seven genes sharing the highest similarity with genes from *X. albilineans*; this contig additionally contains an insertion sequence (IS), which probably explains why the complete LPS cluster could not be assembled in one contig. The occurrence of this IS suggests recent horizontal transfer of LPS genes from *X. albilineans* to *X. campestris* pv. *vasculorum*, both bacterial species spreading in the xylem of sugarcane. Interestingly, the seven LPS-encoding genes exhibiting the highest similarity with *X. albilineans* are involved in sugar metabolism or transport and thus, are thought to be involved in O-antigen biosynthesis. Genes shared by *X. campestris* pv. *vasculorum* and *X. albilineans* may therefore be required for biosynthesis of specific LPSs that are adapted to interactions with sugarcane and are possibly not recognized as PAMPs in this plant. Interestingly, the complete LPS gene cluster is highly conserved between *X. sacchari* and *X. campestris* pv. *vasculorum*. Recently, it was shown that Tn*5*-mutants of *X. albilineans* in XALc_2705 or XALc_2707 were affected in production of disease symptoms and in their capacity to spread within the sugarcane stalk xylem [28].

### Exopolysaccarides

Extracellular polysaccharides (EPS) contribute to the virulence of xanthomonads, in particular by the formation of biofilm [83,84]. The major EPS produced by *Xanthomonas* spp. is called xanthan gum, the production of which is acomplished by a cluster of 12 genes (annotated as *gumB* through *gumM*). *X. fastidiosa* contains nine out of the 12 *gum* genes [85] and the complete cluster is present in *X. sacchari* genome. However, the genome of *X. albilineans* strain GPE PC73 harbors no *gum* gene at all [8]. Furthermore, occurrence of biofilm in the xylem of sugarcane infected by *X. albilineans* has not been reported to date.

*X. albilineans* is the only species of *Xanthomonas* to lack the xanthan gum gene cluster, although this pathogen harbors genes involved in nucleotide sugar biosynthesis using "raw material" during the biosynthesis of carbohydrates found on the bacterial cell surface. Among these genes, some, such as the two key genes *xanA*, encoding a phosphoglucomutase (XALc_2692), and *xanB* encoding a phosphomannose isomerase (XALc_2693), are involved in biosynthesis of xanthan. Other EPS genes, such as *pgi*, *galU* and *ugd*, which encode a glucose-6-phosphate isomerase, an UTP-glucose-1-phosphate uridylyltransferase and an UDP-glucose 6-dehydrogenase, respectively, are also present on the chromosome of *X. albilineans* strain GPE PC73. The role of these genes in biosynthesis of EPS and pathogenicity of *X. albilineans* remains to be investigated. However, it has already been shown that mutation of *xanB* results in an incapacity to produce symptoms and to colonize the sugarcane stalk [28].

Recently, the polysaccharide biosynthesis operon XagABC from *X. campestris* pv. *campestris* was found to be involved in protection against oxidative damage [86]. The *xag* gene cluster may be involved in biofilm formation during the first stage of infection to favor bacterial survival and systemic infection *in planta*. Through quorum sensing during bacterial growth, this cluster is expected to be down-regulated and relayed by up-regulation of *manA* (*xanB* in *X. albilineans*)—a gene that is also involved in biofilm formation in the later systemic stage of infection [86]. Interestingly, the XagABC operon, which occurs in other xanthomonads, was not identified in the genomes of *X. albilineans* and *X. fastidiosa*.

Despite the absence of *gum* genes, the production *in planta* of a xanthan-like polysaccharide by *X. albilineans* strain NCPPB887 has been reported. This polysaccharide, which was purified from diseased sugarcane plants, is formed by repeated tetrasaccharide motifs, each formed by two molecules of glucose, one of mannose, and one of glucuronic acid [87]. The occurrence of glucuronic acid in this xanthan-like polysaccharide was thought to result from the activity of a protease-sensitive UDP-glucose-dehydrogenase. This enzyme, whose N-terminal protein sequence starts with IQPYNH, was purified from sugarcane plants infected by *X. albilineans* strain NCPPB887 [87]. It was hypothesized that *X. albilineans* produces proteases that inhibit this UDP-glucose-dehydrogenase, thus preventing glucuronic acid synthesis and production of the xanthan-like polysaccharide, as observed *in vitro*[87,88]. To explain the production *in planta* of this polysaccharide, it was suggested that glycoproteins produced by sugarcane in response to infection by *X. albilineans* act as powerful inhibitors of proteases synthesized by the pathogen, thus preventing degradation of the UDP-glucose-dehydrogenase and permitting the production of the xanthan-like polysaccharide. As a consequence, this polysaccharide can be produced only in sugarcane plants infected by *X. albilineans*. The genome of *X. albilineans* strain GPE PC73 contains two UDP-glucose-dehydrogenase genes that are conserved in all sequenced *Xanthomonadaceae* (XALc_1655 and XALc_1695), but no gene with an N-terminal or internal IQPYNH sequence could be identified. Further studies are therefore needed to decipher the mechanisms of biosynthesis of this polysaccharide and to confirm its bacterial origin in sugarcane plants infected by the leaf scald pathogen. Indeed, this polysaccharide may be produced by sugarcane to limit spread of *X. albilineans*.

Lack of production of xanthan gum may be a crucial advantage for *X. albilineans*, allowing it to spread within xylem vessels without obstructing them. Complete obstruction of xylem may lead to rapid death of the infected sugarcane and may be unfavorable for *X. albilineans*, which is transmitted mainly by infected cuttings.

### Flagellum and chemotaxis

*X. albilineans* cells, like other *Xanthomonas* spp., carry a single polar flagellum [13]. This flagellum is involved in swimming motility and constitutes a motor organelle that propulses a bacterium through its environment to find optimal conditions *via* chemotaxis (allowing the bacterium to find nutrients or avoid toxic molecules). Chemoreceptors, also called Methyl-accepting Chemotaxis Proteins (MCPs), are associated with the bacterial membrane and are sensitive to various chemical signals to modulate the rotational direction of the flagellum [89,90]. Two sets of genes encoding flagellar assembly (Figure 1) and chemotaxis-related proteins are contiguous in the chromosome of *X. albilineans* strain GPE PC73. The first set of genes, encoding the polar flagellum, shows high homology with the corresponding genes in the other *Xanthomonas* spp. Interestingly, the FliD protein from *X. albilineans* strain GPE PC73 (XALc_1416) harbors a polyserine linker (PSL) that is absent in the corresponding protein encoded by other *Xanthomonas* spp. including *X. sacchari* and also strains NCPPB1131 and NCPPB1132. FliD is the filament-capping protein that forms a cap at the tip of the growing filament structure, which helps the folding process and promotes flagellin subunit insertion and polymerization during helical filament growth [91-93]. FliD is implicated in the virulence of many pathogenic bacteria [94]. In some species, the structural diversity of the cap protein has been reported to be a mean of escaping the host immune system [95]. Indeed, among *Pseudomonas aeruginosa* strains, two distinct FliD proteins were observed, with differences in their primary amino acid sequences resulting in distinct conformations of the flagellum. These two types of FliD proteins differ immunologically. They may play a role in escaping host defenses and, more likely, be responsible for differential binding of strains to respiratory mucins, suggesting adaptability during infections ranging from acute to chronic respiratory infection of cystic fibrosis patients [95]. In *X. albilineans* strain GPE PC73, the presence of a PSL of 12 serine residues in the FliD protein may modify the tertiary structure of the flagellar cap protein when compared with other *Xanthomonas* and, as a consequence, may modify its detection by the sugarcane defense system or be related to a specific interaction with its host.

The second set of genes, encoding chemotaxis-related proteins (from XALc_1353 to XALc_1378), contains five tandemly-repeated genes encoding MCPs, 12 other chemotaxis proteins (Che-like proteins) and six hypothetical proteins (see Additional file 3). In other *Xanthomonas* spp., the number of MCPs is higher, especially in *X. axonopodis* pv. *vesicatoria* (14 tandemly-repeated MCP genes) [30] (Additional file 3). Although the majority of genes encoding chemotaxis receptors proteins are found within a genomic cluster dedicated to chemotaxis and mobility [30,90], some MCP-encoding genes are found scattered on the chromosome. In *X. albilineans* strain GPE PC73, nine additional MCPs were found dispersed on the chromosome (XALc_2151 to 2153, XALc_0649, XALc_0760, XALc_1440, XALc_1926, XALc_2239 and XALc_3131, respectively).

### Bacterial pili or fimbriae

Bacterial pili or fimbriae are proteinaceous multi-subunits structures forming filamentous cell-surface appendages involved in various bacterial virulence processes, including adhesion, biofilm formation, twitching motility, cellular invasion or protein and DNA transport across membranes (DNA uptake during transformation, phage transduction) [96-98]. Like other *Xanthomonas* sequenced to date, the complete genome sequence of *X. albilineans* strain GPE PC73 revealed the presence of a Chaperone-Usher (CU) pilus. Genes involved in fimbriae assembled by the CU-dependent pathway are clustered in an operon together with a periplasmic chaperone (XALc_2025), a predicted outer membrane protein corresponding to the assembly platform called “usher” (XALc_2022) and two candidate structural fimbrial subunits (annotated as hypothetical proteins XALc_2023 and XALc_2021, although they do contain a spore coat U domain also found in pili proteins). These genes share at most 47% overall amino acid identity with their counterparts from other *Xanthomonas* species. Furthermore, like all other sequenced *Xanthomonas*, the genome of *X. albilineans* strain GPE PC73 contains genes able to encode a type IV pilus. Biogenesis of a type IV pilus involves a large number of proteins that are highly conserved within the *Xanthomonas* genus, with the notable exception of the PilA pilin and the PilV-W-X-Y-E operon. The PilA protein is the main structural protein of the type IV pilus. The sequence variability of the *pilA* gene observed among the sequenced *Xanthomonas* could be correlated with host specificity [99]. In *X. fuscans* subsp. *fuscans* strain CFBP4834-R, PilA is involved in adhesion and transmission to seeds, and mutation of *pilA* resulted in reduced pathogenicity on bean [100]. Regarding the PilV-W-X-Y-E operon, a low identity is observed among the whole pool of sequenced xanthomonads. PilQ protein has a kind of intermediate status: it shares over 94% of amino acid sequence identity between all sequenced *Xanthomonas*, with the remarkable exception of *X. albilineans*, the PilQ protein of which showing less than 77% identity. PilQ in *X. oryzae* pv. *oryzae* plays a critical role in virulence, twitching motility and biofilm formation [101], but has no effect on leaf attachment or entry into the host plant [98]. Type IV pilus proteins specific to *X. albilineans* (the PilA pilin and the PilV-W-X-Y-E operon) may be adapted specifically to the xylem of sugarcane. Interestingly, *X. fastidiosa*, which does not possess any flagellum, has both type I and type IV pili at the same pole. Type I (also called Chaperone-Usher), corresponding to the shorter pilus, is involved in adhesion and biofilm formation, whereas the longer type IV pilus is also involved in upstream migration of *X. fastidiosa* against the xylem flow rate [102]. However, the Chaperone-Usher pilus from *X. fastidiosa* seems to differ from that found in xanthomonads. Low sequence conservation and a different gene syntheny suggest that they may play different roles [103].

### Non-fimbrial adhesins

Non-fimbrial (or afimbrial) adhesins are single proteins located on the bacterial cell surface, which are secreted *via* one of three subtypes of the type V secretion system (T5SS) (monomeric autotransporter, trimeric autotransporter and two-partner system [55,104,105]). Non-fimbrial adhesins play a role in attachment and infection processes and, more widely, promote the virulence of phytopathogenic bacteria. Several type V-secreted adhesins are duplicated, frameshifted or truncated in the various sequenced *Xanthomonas* genomes. As a consequence, this genetic variability can lead to misannotated genomes and many predicted adhesins are probably not functional [103,106]. Among all the afimbrial adhesin genes identified in the sequenced *Xanthomonas*, only the following orthologs have been found in the genome of *X. albilineans* strain GPE PC73: XALc_2666 encoding XadA, XALc_2291 encoding an hemolysin protein close to FhaC and XALc_2290 encoding an hemagglutinin/hemolysin protein close to FhaB (followed by XALc_2288 encoding a truncated FhaB form). Moreover, unlike some species of *Xanthomonas* in which a few paralogs of this locus exist that could play specific roles during infection and dissemination [107], *X. albilineans* possesses only one XadA locus. This is illustrated by XadA1 and XadA2 from *X. axonopodis* pv. *phaseoli* in which only paralog XadA2 is involved in vascular transmission to bean seeds [100]. Furthermore, although the genome sequence of *X. albilineans* strain GPE PC73 does not possess a gene encoding the filamentous hemagglutinin YapH that is found in other *Xanthomonas* spp., it encodes two specific non-fimbrial adhesins (XALc_1305 and XALc_1884) matching proteins encoded by *Agrobacterium radiobacter* strain K84 with 54 and 42 percent of amino acid identities, respectively. These non-fimbrial adhesins of *X. albilineans* may play a role in epiphytic survival or in xylem colonization of sugarcane, thus corroborating the adaptation of this pathogen to its specific host and lifestyle. Indeed, a recent study showed that adhesins play a role in the adaptation of xanthomonads to their host plant [107].

### Quorum sensing genes

The *rpf* (for regulation of pathogenicity factors) gene cluster is involved in cell-cell signaling and control of various cellular processes [108]. The *rpf* cluster, first characterized in *X. campestris* pv. *campestris* by [109], comprises nine genes (annotated as *rpfA* through *rpfI*) involved in biosynthesis and detection of DSF (for Diffusible Signal Factor). DSF is a signaling molecule that plays a main role in regulation of the expression of genes required for production of extracellular polysaccharides, biofilm formation and colonization *in planta*[110]. DSF biosynthesis is governed mainly by *rpfF*—a gene encoding an enoyl-CoA hydratase—whereas both *rpfC* (encoding a hybrid two-component DSF sensor) and *rpfG* (encoding a two-component regulator) are implicated in DSF perception and signal transduction, respectively. The *rpf* genes are unique and specific to the xanthomonads *X. fastidiosa* and *S. maltophilia*[90,111], although a *Burkholderia cepacia* gene showing 37% identity at the peptide level with *rpfF* from *X. campestris* pv. *campestris* has been shown recently to be involved in biosynthesis of a DSF functional analog called BDSF [111]. In *X. albilineans* and *X. fastidiosa, rpfD*, *rpfH* and *rpfI* are missing. Similarly, *X. axonopodis* pv*. citri* does not possess *rpfH* and *rpfI*[31]. The RpfH protein is related structurally to the sensory input domain of RpfC [112]. The *rpfD* gene encodes a transcriptional regulator and *rpfI* encodes a protein that positively regulates the biosynthesis of proteases, endoglucanases and EPS in *X. campestris* pv. *campestris*[113]. Even if the *rpf* gene cluster appears to be incomplete, it plays a key role in pathogenicity and insect transmission of *X. fastidiosa*[27,114]. DSF is also produced by *X. albilineans*[115], and the importance of this molecule in the pathogenicity of this species is currently under investigation. Studying the regulatory Rpf/DSF pathway by comparing gene expression in the wild type strain *versus* an *rpf* mutant may be a promising approach with which to identify pathogenicity-related genes of *X. albilineans*.

### Two-component signal transduction systems

The genomes of *Xanthomonas* spp. encode numerous two-component signal transduction systems (TCSTSs). These molecular sense-response mechanisms are able to act to regulate the expression of genes involved in cell motility, virulence, biofilm formation, chemotaxis, quorum-sensing and many others cellular processes (e.g. [116,117]). It has been suggested that the number of these signaling proteins occurring in the genome reflects the adaptation of xanthomonads to stochastic environmental changes and heterogeneous ecological niches [116,118]. We identified 98 two-component system proteins in the chromosome of *X. albilineans* strain GPE PC73 (Additional file 4). These proteins include 35 two-component system sensor proteins, 45 two-component system regulatory proteins and 18 two-component system sensor-response regulator hybrid proteins. The number of TCSTSs identified in *X. albilineans* is lower than that found in *X. axonopodis* pv. *vesicatoria* strain 85–10 (121 genes) but higher than the number found in *X. oryzae* pv. *oryzae* strain KACC10331 (92 genes) [116].

### Transposases

Transposases catalyze the transposition mechanisms of mobile genetic elements. They are involved in DNA rearrangement and play a role in both genome evolution and cellular function [119]. The chromosome of *X. albilineans* strain GPE PC73 harbors 86 transposases or transposase fragments (Additional file 5). This number is similar to those reported for other *Xanthomonas* spp. with the notable exception of *X. oryzae* pv. *oryzae* and *X. oryzae* pv. *oryzicola* strains, which possess a very high number of insertion sequences (IS) covering 20% of the genome [120]. Twenty-two transposases of *X. albilineans* are unique to this pathogen, while the others are shared or partially shared by many *Xanthomonas* species. However, the open reading frames XALc_2324, XALc_2365, XALc_2622 and XALc_2931 match only with transposase sequences from the vascular rice pathogen *X. oryzae* pv. *oryzae*. Interestingly, transposases encoded by XALc_1244 and XALc_2604 were found only in two sugarcane pathogens (*X. albilineans* and *X. campestris* pv. *vasculorum*) and in a banana pathogen (*X. campestris* pv. *musacearum*) that is closely related to one of these sugarcane pathogens [32].

### A 5-kb gene cluster present in 12 different locations of the chromosome

An intriguing genomic feature of *X. albilineans* strain GPE PC73 is the presence of 12 similar, but not identical, copies of a 5-kb gene cluster in 12 locations of the chromosome of strain GPE PC73 (Figures 1 and 2). Most of these genes are not conserved in all 12 copies, but some genes conserved in several copies share high percentages of overall amino acid identity (Figure 2), suggesting that they are involved in similar molecular processes. Proteins encoded by these 5-kb gene clusters, annotated as hypothetical proteins, do not contain any conserved functional domain and match only partially with hypothetical proteins from *X. campestris* pv. *musacearum*, *X. campestris* pv. *vasculorum* or *X. oryzae* pv. *oryzae*. Although these proteins are not found in the genome of *X. sacchari* strain NCPPB4393, two of them (XALc_1969 and XALc_1970) are partially conserved in a single copy in the genome of *Xanthomonas* spp. NCPPB1131.

The 12 copies of the gene cluster each encode a protein harboring the triade Ser-His-Asp (SHD) characteristic of some members of the α/β-hydrolase fold enzyme superfamily. This very large group of hydrolytic enzymes exhibits a catalytic site involving a nucleophilic amino acid (serine or cysteine) within a Gx(S/C)xG signature sequence, a histidine and an acidic amino acid (aspartic acid or glutamic acid) [121]. These three catalytic residues are dispersed in the primary sequence, but the 3D-structure of the enzyme brings them together to form the catalytic site. In *X. albilineans*, the signature sequence is GxSxG, and all the catalytic triades are from the SHD form found in thioester hydrolases, serine carboxypeptidases, haloperoxydases, hydroxynitrile lyases, C-C bond hydrolases and lipid hydrolases. Moreover, all SHD triades found in the *X. albilineans* genome are organized in almost the same way as lipases from *Pseudomonas fluorescens*[122].

Additionally, nine proteins harboring the triade SHD also contain a polyserine linker (PSL) that separates two different putative functional domains of unknown functions. Similar PSLs have been described as flexible spacer regions that enhance substrate accessibility, thus facilitating the enzymatic activities of proteins [70]. The presence of a PSL and/or the SHD triade, may indicate that the 12 copies of the 5-kb gene cluster are involved in molecular processes required for the use of substrates specifically present in sugarcane sap. Five copies of this gene cluster contain a *virD4*-like gene encoding a NTPase which may be required to power such specific molecular processes. The 12 proteins containing a PSL and/or the triade SHD, each present in one copy of the cluster, share some amino acid similarity with each other, but they can be clustered in three distinct groups according to their amino acid identity. XALc_1969, XALc_2380, XALc_1070, XALc_3014, XALc_2331, XALc_2613 and XALc_2074-75 share 59–88% amino acid identity. XALc_3056 and XALc_0685 share 93% amino acid identity. XALc_1798, XALc_1863 and XALc_1627 share at least 84% amino acid identity. The three groups share together less than 45% amino acid identity, indicating that they may be involved in three distinct molecular processes. Nine copies of this 5-kb gene cluster are flanked by phage and/or recombination hot spot (Rhs) sequences, indicating that the 12 copies of this 5-kb gene cluster were probably acquired by lateral gene transfer.

### Clustered regularly interspaced short palindromic repeats systems (CRISPRs)

CRISPRs are repetitive structures in bacteria and Archaea composed of exact 24- to 48-bp repeated sequences separated by unique spacers of similar length. Over 40 gene families, which are found nowhere except near these repeats, have been designated collectively as CRISPR-associated (*cas*) genes [123,124]. CRISPR/*cas* systems belong to different classes, with different repeat patterns or sets of genes, and are distributed widely in a large range of species. Recent data showed that CRISPR/*cas* systems participate in an antiviral response, probably by an RNA interference-like mechanism [125]. CRISPR interference may occur when the spacer sequences within CRISPR/*cas* systems match with corresponding bacteriophage or plasmid sequences. We identified two different CRISPR/*cas* systems in the genome of strain GPE PC73 of *X. albilineans* (Figure 1). The first system, CRISPR-1, is similar to that found in *X. oryzae* pv. *oryzae*, *X. axonopodis* pv. *citri, X. campestris* pv. *vasculorum* and *X. campestris* pv. *musacearum.* This system is associated with seven *cas* genes: *cas3* (XALc_2885), *cas5d* (XALc_2887), *csd1* (XALc_2888), *csd2* (XALc_2889), *cas4* (XALc_2890), *cas1* (XALc_2891), *cas2* (XALc_2892), and contains thirty-four 31-base pair repeats and thirty-three 33- to 38-base pair spacers. The number of spacers in CRISPR-1 in *X. albilineans* strain GPE PC73 is lower than that in *X. oryzae* pv. *oryzae* (37–77 spacers depending on sequenced strains [126]). The second system, CRISPR-2, is associated with six *cas* genes: *cas1* (XALc_3048), *cas3* (XALc_3049), *csy1* (XALc_3050), *csy2* (XALc_3051), *csy3* (XALc_3052), *csy4* (XALc_3053), and contains twenty-four repeats of 28 base pairs and twenty-three spacers of 32 base pairs. There is only one other *Xanthomonas* pathovar that is known to contain a similar CRISPR-2 system, namely *X. campestris* pv. *raphani*[34]. Interestingly, spacers of CRISPR-1 and CRISPR-2 of *X. albilineans* strain GPE PC73 are identical to the phage-related DNA sequences present only in the chromosome of this strain (prophage; Additional file 6), indicating the existence of an hitherto unknown world of *X. albilineans*-specific bacteriophages. Additionally, orthologs of some ORFs encoded by one of these phage-related sequences are present in *X. fastidiosa* strain 9a5C (XALc_0178 = XF0935, XALc_0206 = XF0508, XALc_0209 = XF0510 and XALc_1544 = XF1859). Only one CRISPR spacer matches a non phage-related gene (XALc_0969 encoding a dCTP deaminase, Additional file 6). The presence of two CRISPR systems may indicate that *X. albilineans* is adapted to live in environments containing phages. Many endophytic and pathogenic bacteria have been isolated from sugarcane xylem [127]. Resistance to phages may confer a competitive advantage to *X. albilineans* cohabiting in sugarcane xylem vessels with rival bacteria that might produce phages. Resistance to phages may also be required to survive in environments other than xylem vessels (rain water, leaf surfaces…) during aerial spread of *X. albilineans*[16].

### Putative pathogenicity-related genes identified by comparison by suppression subtractive hybridization (SSH) of two strains of *X. albilineans* differing in pathogenicity

**Figure 4:**
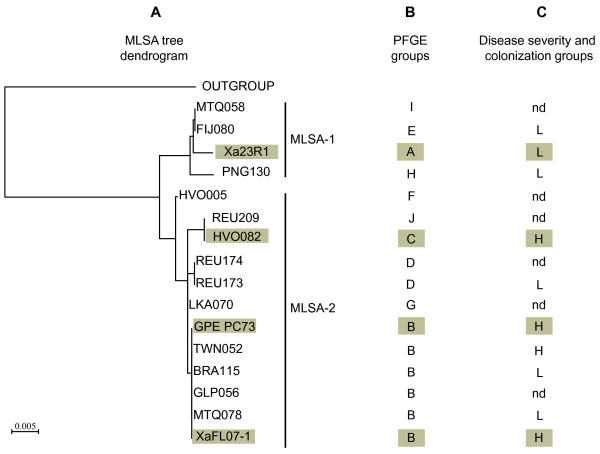
**Phylogenetic analyses of 16 strains of *X. albilineans *spanning the genetic diversity of this species and belonging to different PFGE groups: A: MLSA dendrogram obtained with 16 strains of *X. albilineans *from diverse geographical origins and rooted with *X. albilineans *strain XaS3 (outgroup).** Strain XaS3 was isolated in Guadeloupe from the surface of a sugarcane leaf and failed to induce symptoms in sugarcane after plant inoculation [16]. The tree was constructed with concatenated sequence of the PCR fragments of seven housekeeping genes (*gyrB, groEL, atpD, dnaK, efp, glnA* and *recA*) using the maximum likelihood method and GTR as substitution model. Strains on a grey background correspond to strains Xa23R1, HVO082, GPE PC73 and XaFL07-1 used for SSH comparative *in silico* analysis. **B:** PFGE groups assigned to each *X. albilineans* strain, data from Davis et al. (1997) [24] and Rott P. (unpublished data). PFGE groups on a grey background are groups corresponding to strains Xa23R1, HVO082, GPE PC73 and XaFL07-1 used for SSH comparative *in silico* analysis. **C:** Pathogenicity in sugarcane rated as disease severity and extent of stalk colonisation (data from [25,130]; Rott P, unpublished data): H = highly aggressive group, L = low or nonaggressive group and nd = no data. Groups on a grey background are groups corresponding to strains Xa23R1, HVO082, GPE PC73 and XaFL07-1 used for SSH comparative *in silico* analysis

A large diversity exists among strains of *X. albilineans*; ten genetic groups (PFGE-A to PFGE-J) have been identified by PFGE analyses [24] Rott et al., personal communication). Additionally, disease severity and capacity to colonize sugarcane stalks also varies between strains of *X. albilineans*[16,25,128,129]. As a first step towards establishing the phylogenetic relationship between the different PFGE-groups, we performed a multi-locus sequence analysis (MLSA) using a set of seven housekeeping genes fragments (*gyrB, groEL, atpD, dnaK, efp, glnA* and *recA*) with 16 strains spanning the diversity of *X. albilineans* (Table 1). The dendrogram obtained using the maximum likelihood method proposed a phylogeny in which PFGE groups are clustered in two main distant clades called MLSA-1 and MLSA-2 (Figure 4). The robustness of the tree topology clustering PFGE groups in these two main clades was confirmed by high bootstrap values (86% and 100%). MLSA-1 contains four PFGE groups and MLSA-2 contains six PFGE groups. Interestingly, all highly pathogenic strains are grouped in clade MLSA-2. To further survey genes required for pathogenicity of *X. albilineans* strains, we performed suppression subtractive hybridization (SSH) to compare the genome of a highly pathogenic strain (XaFL07-1) belonging to clade MLSA-2 with a less pathogenic strain (Xa23R1) belonging to clade MLSA-1. Both strains originate from Florida. Their respective genome sequences are not available to date. Strain XaFL07-1 was recently used to identify new putative pathogenicity genes by transposon mutagenesis [28], and strain Xa23R1 was used in a previous study to clone the albicidin biosynthesis gene cluster [64]. The 42-kb genomic region of strain Xa23R1 encoding the T3SS SPI-1 was also sequenced recently [48]. Interestingly, strains XaFL07-1 and Xa23R1 belong to PFGE groups B and A, respectively. Davis et al. (1997) [24] previously showed in a greenhouse experiment that strains of *X. albilineans* belonging to genetic group B are able to infect a higher number of stalks than strains of group A after inoculation of sugarcane plants by the decapitation technique. Additionally, strains of group B are spread aerially whereas strains of group A are not. 

**Table 1:** **Characteristics of *Xanthomonas albilineans *strains from worldwide locations used in this study**

**Strain**	**Origin**	**Year**	**Author**	**Haplotype PFGE**^**x**^	**Clade MLSA**^**y**^
HVO005	Burkina Faso	1980	M. Chatenet	PFGE-F	2
TWN052	Taïwan	1988	C.T. Chen	PFGE-B	2
GLP056	Guadeloupe	1988	P. Rott	PFGE-B	2
MTQ058	Martinique	1989	P. Rott	PFGE-I	1
LKA070	Sri Lanka	1962	A.C. Hayward	PFGE-G	2
MTQ078	Martinique	1957	J.A. Spence	PFGE-B	2
FIJ080	Fiji	1961	D.W. Dye	PFGE-E	1
HVO082	Burkina Faso	1989	M. Granier	PFGE-C	2
PNG130	Papua-New Guinea	1993	M. Chatenet	PFGE-H	1
BRA115	Brazil	1993	C.O.N. Cardoso	PFGE-B	2
GPE PC73	Guadeloupe	2003	P. Champoiseau	PFGE-B	2
REU173	Reunion Island	1995	J-C. Girard	PFGE-D	2
REU174	Reunion Island	1995	J-C. Girard	PFGE-D	2
REU209	Reunion Island	1995	J-C. Girard	PFGE-J	2
Xa23R1	Florida/USA	1993	M.J. Davis	PFGE-A	1
XaFL07-1	Florida/USA	2007	P. Rott	PFGE-B	2

^x^[24]; Rott et al. personal communication.

^y^ This study: MLSA (Multi Locus Sequence Analysis).

We enriched a library of unique DNA sequences from strain XaFL07-1 (tester strain) using Xa23R1 DNA as the driver strain. A total of 143 XaFL07-1-specific clones were generated and sequenced. Sequences were compared with the genome sequences of two highly pathogenic *X. albilineans* strains, *i.e.* strain GPE PC73 which belongs to the same PFGE-B group as strain XaFL07-1, and strain HVO082, which belongs to group PFGE-C. Strains XaFL07, GPE PC73 and HVO082 all belong to clade MLSA-2 (Figure 4). We used the complete genome sequence of strain GPE PC73 available in Genbank and an unpublished draft genome sequence of strain HVO082 (Rott P. et al., unpublished data). Within the library of 143 clones, 50 clones were absent in the genome of strains GPE PC73 and HVO082, and were consequently considered as specific to strain XaFL07-1. Twenty-five clones targeting the same ORF as another clone were considered as duplicates. The occurrence of these duplicates reflected the good coverage of the genome and reliability of the method used to construct the SSH library. The remaining 68 SSH fragments were present in strain GPE PC73, but only 18 of them occurred also in strain HVO082. These 18 SSH fragments were therefore considered specific to the three highly pathogenic strains (XaFL07-1, GPE PC73 and HVO082).

The 18 SSH fragments correspond to 20 annotated ORFs and to one large intergenic sequence (2,117 bp) of strain GPE PC73 (Table 2). This large intergenic sequence, specific to the three highly pathogenic strains, is bordered in strain GPE PC73 by one tRNA (XALc_0254) and an entericidin gene conserved in all other sequenced xanthomonads (XALc_0255). Among the 20 ORFs that were absent in the less pathogenic strain (Xa23R1) and present in three highly pathogenic strains, five of them are in the same genomic region: they encode an ATP-binding cassette (ABC) transporter (XALc_1232 and XALc_1231) and a hypothetical protein (XALc_1229) which are conserved in *X. oryzae* pv. *oryzicola* BLS256, and two hypothetical proteins (XALc_1226 and XALc_1228) specific to *X. albilineans* (Table 2). Two other ORFs specific to highly pathogenic strains encode an ABC transporter specific to *X. albilineans* (XALc_0703 and XALc_0704). One ORF, which encodes a hypothetical protein, and which is specific to highly pathogenic strains, is present in strain GPE PC73 in a region encoding ABC transporters (XALc_2561), suggesting that it may also be involved in such a function. ABC transporters specific to highly pathogenic strains could contribute to pathogenicity by secretion of small molecules or proteins or, alternatively, could confer resistance against toxins produced by the sugarcane plant. Two additional ORFs with an assigned function that are specific to the three highly pathogenic strains are also candidate pathogenicity genes based on their predicted function. One encodes a methyl-accepting protein (XALc_1361) belonging to the flagellum gene cluster, which may be required in response to temporal changes in the chemical environment during colonization of the sugarcane xylem vessels. The other encodes an oxidoreductase (XALc_2283) that could protect the pathogen from the oxidative burst resulting from recognition of *X. albilineans* by the sugarcane plant. Interestingly, the reciprocal best BLAST hit in GenBank for this ORF belongs to *X. campestris* pv. *vasculorum*. This ORF is also conserved in strain ATCC35937 of *X. axonopodis* pv. *vesicatoria*, but is not conserved in any other sequenced xanthomonads.

**Table 2:** SSH clones and targeted ORFs present in highly pathogenic strains (XaFL07-1, GPE PC73 and HVO082)

**XaFL07-1 SSH clone**	**GPE PC73 ORF**	**Protein size**	**Protein description/predicted Pfam domain or predicted function**	**Paralog (% identity in Aa)**	**Best Blast Hit with other xanthomonads (NCBI)**
VI-G9	XALc_1622	540 Aa	Hypothetical protein/Integral membrane sensory domain found in signaling proteins	XALc_2416 (83%)	No hit found
I-C4	XALc_2283	351 Aa	Hypothetical protein/Cytochrome P450 superfamily (oxidoreductase)		XVA NCPPB702: XcampvN_010100019130 with S:1228, E: 1e-167, I: 244/347 (70%)
I-C4	XALc_2284	35 Aa	Hypothetical protein/Pseudogene		No hit found
I-D2	XALc_2627	87 Aa	Hypothetical protein/Phage	XALc_2395 (99%)	No hit found
I-D2	XALc_2628	168 Aa	Hypothetical protein/Phage	XALc_2396 (98%)	No hit found
I-F8	XALc_1545	69 Aa	Hypothetical protein/Phage		No hit found
I-F8	XALc_1546	72 Aa	Hypothetical protein/Phage		No hit found
I-G2	XALc_2561	223 Aa	Hypothetical protein/Unknown (located near ABC transporter genes)		XOO MAFF 311018: XOO_1679 with S:1092, E: 4e-151, I: 209/223 (94%)
III-E3	XALc_0703	311 Aa	ABC transporter		No hit found
V-H6	XALc_0704	400 Aa	Hypothetical protein/ABC transporter		No hit found
III-H5	XALc_1631	254 Aa	Hypothetical protein/Phage		No hit found
IV-B5	XALc_1361	847 Aa	Methyl-accepting chemotaxis protein		XCR 756C: XCR_213 with S:2630, E: 0, I: 533/798 (67%)
IV-B12	XALc_1803	211 Aa	Hypothetical protein/Phage		XCV 85-10: XCV2218 with S:383, E:9e-44, I:96/199 (48%)
VI-A11	XALc_2887	224 Aa	CRISPR-associated protein Cas5d		XOO MAFF 311018: XOO_0797 with S:1041, E: 2e-143, I: 195/222 (88%)
VI-H8	XALc_2384	462 Aa	Recombinase/Resolvase (Phage)	XALc_2617 (97%)	No hit found
II-H2	XALc_1232	415 Aa	ABC transporter		XOC BLS256: Xoryp_010100020185 with S:1998, E:0, I:388/415 (93%)
III-F5	XALc_1231	439 Aa	ABC transporter		XOC BLS256: Xoryp_010100020190 with S:2041:, E:0, I:397/439 (90%)
V-F1	XALc_1228	413 Aa	Hypothetical protein/Unknown		No hit found
V-F1	XALc_1229	272 Aa	Hypothetical protein/Unknown		XOC BLS256: Xoryp_20200 with S:1261, E:8e-175, I:240/273 (88%)
V-H8	XALc_1226	198 Aa	Hypothetical protein/Unknown		No hit found
VI-H11	Intergenic Sequence	2,117 bp	located between a tRNA (XALc_0254) and an entericidin gene (XALc_0255)		

XCV: *X. campestris* pv. v*esicatoria;* XOO: *X. oryzae* pv. *oryzae;* XCR: *X. campestris* pv. *raphani;* XOC: *X. oryzae* pv. *oryzicola;* XVA: *X. campestris* pv. *vasculorum*

bp: nucleotide base pair; Aa: amino acid; S: score; E: E value; I: % of identity between query sequence and subject sequence.

Finally, one ORF, which encodes a hypothetical protein containing an integral membrane sensory domain, and which is specific to highly pathogenic strains (XALc_1622), is present in two copies in the genome of strain GPE PC73. The remaining ORFs are not considered as putative pathogenicity genes. The smallest ORF (XALc_2284) is probably a pseudogene deriving from an ancestral copy of XALc_2292 (RNA methylase). The recombinase-resolvase (XALc_2384) probably belongs to a phage sequence. The remaining hypothetical protein genes specific to highly pathogenic strains are bordered by phage-related sequences and probably also belong to a phage sequence (XALc_2627, XALc_2628, XALc_1545, XALc_1546, XALc_1631 and XALc_1803). The absence in strain Xa23R1 of the locus CRISPR-1 was confirmed by PCR using several pairs of primers specific of this locus. This result suggests that strain Xa23R1 may be less adapted to life in phages-containing environments than highly pathogenic strains.

## Conclusions

In this study, we present a comparative genomic analysis of the complete genome sequence of the highly pathogenic strain GPE PC73 of *X. albilineans*. This *in silico* analysis revealed genomic features specific to *X. albilineans* that may play key roles in strategies used by this pathogen to spread within sugarcane xylem vessels with a reduced artillery compared to other pathogenic *Xanthomonas* species.

The most important strength of *X. albilineans* is that it is very well equipped for spread in a nutrient-poor environment. In this respect, the 35 TBDT genes found on the chromosome of *X. albilineans* strain GPE PC73 could play a key role in the active transport of plant nutrients. Interestingly, two TBDT genes (XALc_0643 and XALc_0723) were previously identified to contribute to disease severity and extent of stalk colonization [28]. The five cellulases exhibiting a CBD and a long linker region (XALc_2967, XALc_2969, XALc_0484, XALc_0865 and XALc_0874) may be adapted specifically to use cell breakdown products as carbon sources. The presence of 12 copies of a 5-kb gene cluster encoding hypothetical proteins, which are predicted to be involved in the degradation of nutrients because they harbor a PSL or a lipase-specific triade, may also be linked to the adaptation to the poor nutrient conditions prevailing in the xylem of sugarcane. Additionally, life in the iron-poor xylem sap may require unknown siderophores that are possibly synthesized by the new NRPS genes identified in the genome of strain GPE PC73 of *X. albilineans*.

In the rather cloistered environmental niche inside xylem vessels, *X. albilineans* most likely uses strategies to avoid detection by plant defense systems. The presence of a PSL in the FliD protein might help mask the bacterium from the sugarcane's defense surveillance. LPS genes shared with *X. campestris* pv. *vasculorum*, another sugarcane-interacting species, might be involved in biosynthesis of specific LPS that are poorly recognized as PAMP by sugarcane surveillance systems. Similarly, the reductive genome evolution experienced by *X. albilineans* during its descent from the common ancestor of *Xanthomonas* may have favored an adaptation to sugarcane xylem vessels by allowing the loss of genes encoding PAMPs recognized by sugarcane surveillance systems.

As shown in other plant-microbe interactions, *X. albilineans* most likely uses various strategies to protect itself against sugarcane defense systems. ABC transporters specific to highly pathogenic strains may confer resistance against antibacterial toxins produced by sugarcane. The gene XALc_1762, which encodes the membrane fusion of an MDR efflux pump, may be involved in a similar resistance process because it was shown previously to confer resistance to novobiocin and to be required for efficient stalk colonization of sugarcane by *X. albilineans*[28]. Interestingly, another ABC transporter (XALc_0028) was shown previously to be required for spreading within sugarcane [28]. Additionally, three ABC transporter proteins from *X. albilineans* GPE PC73 (XALc_941, XALc_942 and XALc_944) have also been identified in the genome of *Leifsonia xyli* subsp. *xyli*, *Acidovorax avenae* subsp. *avenae* and *X. sacchari*, respectively, which all are able to spread within xylem vessels of sugarcane, making these ABC transporter proteins also good candidates for future studies (orthoMCL analysis; Pieretti I., personal communication). Additionally, the oxidoreductase specific to highly pathogenic *X. albilineans* strains may play a role by protecting the bacteria from the oxidative burst resulting from the recognition of *X. albilineans* by the sugarcane plant. Another oxidoreductase (XALc_1972) that has been shown as shared only by *X. albilineans* strain GPE PC73, *X*. sacchari and *L. xyli* subsp. *xyli* may have a similar function*.* TBDTs could also play a key role in the detoxification of antibacterial toxins produced by sugarcane that target external components of *X. albilineans* by allowing their active and fast uptake inside the bacterium.

Additionally, *X. albilineans* may use specific and unknown strategies to suppress or weaken sugarcane plant defenses. Because neither a T3SS-Hrp nor the corresponding effectors have been identified in the genome of *X. albilineans*, the pathogen might rely on other secretion systems identified in the genome of *X. albilineans* strain GPE PC73. Secretion systems T1SS and T2SS might possibly be involved in the secretion of yet-to-be identified effectors that could be secreted in the xylem and diffuse to the parenchyma target cells. Potentially, such effectors may be synthesized by the new NRPS genes identified in the genome of strain GPE PC73 of *X. albilineans*. Some of the 216 hypothetical protein genes specific to *X. albilineans* may be required to suppress or weaken sugarcane plant defenses. However, screening of a random mutant library of strain XaFL07-1 of *X. albilineans* failed to identify any pathogenicity gene predicted to be involved in an offensive or counter-defensive mechanism [28].

To conclude, neither comparative genomic analyses nor SSH experiments have revealed the existence of genes involved in any offensive or counter-defensive mechanism specific to *X. albilineans*, at least unless the role of hypothetical proteins can be determined, suggesting that this pathogen is able to spread within sugarcane xylem vessels with only a reduced artillery. This is even more surprising with respect to preliminary confocal microscopy data, which revealed recently that highly pathogenic strains XaFL07-1 and GPE PC73 of *X. albilineans* are able to spread efficiently in the xylem and also in other leaf and stalk tissues (Mensi I., personal communication).

## Methods

### Bacterial strains and media

The origin and main characteristics of the bacterial strains studied in this paper are summarized in Table 1. *X. albilineans* strains Xa23R1 and XaFL07-1 stored at −80°C were retrieved by plating on Wilbrink’s agar medium (peptone 5 g, yeast extract 5 g, sucrose 10 g, sodium sulfite (Na_2_SO_3_, 7 H_2_O) 0.05 g, magnesium sulfate (MgSO_4_, 7 H_2_O) 0.25 g, dipotassium phosphate (K_2_HPO_4_) 0.5 g, Benomyl® 12.5 mg, distilled water 1 l, pH 6.9-7.0).

### Genome sequence of *X. albilineans* strain GPE PC73

*X. albilineans* strain GPE PC73 is referred to as CFBP 7063 in the French Collection of Plant Pathogenic Bacteria (http://www.angers.inra.fr/cfbp/). The complete annotated genetic map, search tools (BLAST), annotation and classification process are available at [http://iant.toulouse.inra.fr/X.albilineans].

### Draft genome sequence of *X. albilineans* strain HVO082

*X. albilineans* strain HVO082 from the Bacterial Collection of UMR BGPI at Cirad in Montpellier, France, was sequenced recently by Genoscope (Evry, France) (Rott P., unpublished sequence).

### Extraction of bacterial genomic DNA

Bacterial genomic DNA was extracted from 5 ml of culture grown in liquid Wilbrink’s medium for 48 h at 28°C with the Illustra GenomicPrep Bacteria Mini Spin Kit (GE Healthcare, Buckinghamshire, UK), following the manufacturer's instructions.

### Construction of suppression subtractive hybridization librairies

Genomic DNA was extracted from *X. albilineans* strains GPE PC73 and XaFL07-1 as described above. The subtracted DNA library was generated following the instructions with the PCR-Select Bacterial Genome Subtraction Kit (Clontech Laboratories, Inc., Mountain View, CA, USA). Approximately 2 μg of both XaFL07-1 and Xa23R1 genomic DNA were digested with *Hae*III for 5 h at 37°C with 25 units of enzyme. *Hae*III-digested DNA from XaFL07-1 was used as the tester and Xa23R1 was used as the driver for XaFL07-1 library construction. Two different adaptors (1 and 2R) were ligated to the 5’ end of two aliquots of the tester DNA fragments. Two hybridizations were performed. In the first, the driver DNA fragments were added to each adaptor-ligated tester DNA (ratio 2:1). Then, the DNA fragments were denatured for 90 s at 98°C and allowed to anneal at 63°C for 1.5 h. The two samples (one with adaptor 1 and the other with adaptor 2R) were mixed together and freshly denatured driver DNA was added before the second hybridization. The second hybridization was carried out for 16 h at 63°C. Two sequential nested PCR reactions were carried out. The subtraction efficiency was measured by PCR amplification of the *rpfB* gene, present in these two strains and which does not contain any *Hae*III restriction site. The *rpfB* gene was amplified using rpfB-forward (CTCTGGTGGAAGCCTACGGAC) and rpfB-reverse (CAATACGCCTGGCATCATCGC) primers, under the PCR conditions of the supplier. Five μl of amplimers were removed after 18, 21, 24 and 27 PCR cycles, respectively, and examined by agarose gel electrophoresis. The difference in the number of cycles required for equal amplification of the corresponding PCR product in the subtracted and unsubtracted samples indicated that the efficiency of our subtraction was six. According to the supplier’s instructions, six cycles corresponds roughly to a 12-fold enrichment.

The PCR products were cloned into the pCR®8/GW/TOPO® TA Cloning® vector system (Invitrogen, Carlsbad, CA, USA). The SSH library was constructed by transforming the cloned vector into One Shot® TOP10 Chemically Competent *E. coli* cells according to the supplier’s instructions (Invitrogen, Carlsbad, CA, USA). For each colony, bacterial suspension samples after dilution were amplified by PCR using T7 and M13 forward primers to confirm the presence and size of the different inserts by agarose gel electrophoresis.

### Dot blot screening

Each PCR clone was subjected to differential dot blotting to identify those that hybridized preferentially to the tester XaFL07-1 DNA. Briefly, 2 μl of each denatured PCR sample were dotted in duplicate on positively-charged nylon membranes (Roche, Mannheim, Germany). DNA fixation was carried out by irradiation under a UV transilluminator for 2 min. A digoxigenin-11-2'-dUTP DNA labeling and detection kit (Roche, Mannheim, Germany) was used to prepare the probes. The genomic DNA of both strains, Xa23R1 and XaFL07-1, were used independently as a probe for each of the duplicate membranes. Prehybridization and hybridization were carried out according to the manufacturer's instructions (Roche, Mannheim, Germany).

### DNA sequencing and analysis

Specific inserts were sequenced using T7 and M13 forward sequencing primers. Sequencing reactions were performed using the Big Dye kit (Applied Biosystems, Foster City, CA, USA) and were analysed on an Applied Biosystems 3100 automated DNA sequencer by CGRC/ICBR (University of Florida, Gainesville, FL, USA). Sequence assembly and further editing were performed with Bioedit Analysis software (Tom Hall, Carlsbad, CA, USA). Blastx sequence homology analyses were performed using the Basic Local Aligment Search Tool (BLAST) [131] network service of the National Center for Biotechnology Information (NCBI) and using iANT (integrated ANnotation Tool) [132].

### Genomic comparison

For comparative analyses, the annotated genome sequences of *X. albilineans* strain GPE PC73, GenBank accession no. NC_013722.1 [8] was compared to the unpublished draft sequence of strain HVO082 of *X. albilineans* (Rott P., unpublished data) and to seven published genome sequences of *Xanthomonas*: *X. axonopodis* pv. *vesicatoria* strain 85–10, GenBank accession no. NC_007508.1 [30], *X. axonopodis* pv. *citri* strain 306, GenBank accession no. NC_003919.1 [31], *X. campestris* pv. *campestris* strain ATCC 33913, GenBank accession no. NC_003902.1 [31], *X. campestris* pv. *vasculorum* NCPPB702, GenBank accession no. NZ_ACHS00000000.1 [32], *X. campestris* pv. *musacearum* NCPPB4381, GenBank accession no. NZ_ACHT00000000.1 [32], *X. oryzae* pv. *oryzae* MAFF 311018 [33] and *X. oryzae* pv. *oryzicola* strain BLS256, GenBank accession no. CP003057 [34]. Homology searches were conducted at the nucleotide and amino acid sequence levels using the BLAST server [131].

### OrthoMCL analysis

OrthoMCL clustering analyses [133] were performed using the following parameters: P-value Cut-off = 1 × 10^-5^; Percent Identity Cut-off = 0; Percent Match Cut-off = 80; MCL Inflation = 1.5; Maximum Weight = 316. We modified OrthoMCL analysis by inactivating the filter query sequence during the BLASTP pre-process. Best BLASTP hit analyses were performed with database UniProt by excluding all accessions from the xanthomonads using expectation value lower than 1 × 10^-5^.

### Phylogenetic analysis

The phylogenetic MLSA tree was reconstructed using the maximum likelihood method implemented in the PhyML program. The GTR substitution model was selected assuming an estimated proportion of invariant sites (of 0.01) and four gamma-distributed rate categories to account for rate heterogeneity across sites. The gamma shape parameter was estimated directly from the data (α= 0.010). Five hundred bootstrap replicates were performed with the PhyML program. The seven analyzed loci (*gyrB*, *groEL*, *recA*, *dnaK*, *efp*, *atpD*, and *glnA*) are typical housekeeping genes in the chromosome of *X. albilineans* GPE PC73, located 0.004, 0.348, 1.369, 1.983, 2.245, 3.442, and 3.655 Mb, respectively, from the origin of replication. The total length of the partial CDS nucleotide sequences concatenated for each taxon was 4,016 bp. Multiple alignments of the nucleotide sequences of the seven housekeeping fragment genes and for all taxa were performed using ClustalW.

## Abbreviations

ABC: ATP-binding cassette; BLAST: Basic Local Alignment Search Tool; CBD: Cellulose Binding Domain; CRISPRs: Clustered Regularly Interspaced Short Palindromic Repeats systems; CU: Chaperone-Usher; DSF: Diffusible Signal Factor; Hrp: Hypersensitive Response and Pathogenicity; IS: Insertion Sequence; LPS: Lipopolysaccharides; MCP: Methyl-accepting Chemotaxis Proteins; MDR: Multidrug resistance; MLSA: Multilocus sequence analysis; NCBI: National Center for Biotechnology Information; NRPS: Nonribosomal peptide synthetases; PAMP: Pathogen-Associated Molecular Pattern; PFGE: Pulsed-Field Gel Electrophoresis; PSL: Polyserine linker; Rhs: Recombination hot spot; rpf: Regulation of pathogenicity factors; SPI-1: *Salmonella* Pathogenicity Island-1; SSH: Suppression Subtractive Hybridization; T1SS: Type I Secretion System; T2SS: Type II Secretion System; T3SS: Type III Secretion System; T4SS: Type IV Secretion System; T5SS: Type V Secretion System; T6SS: Type VI Secretion System; TBDT: TonB-Dependent Transporters; TCSTS: Two-Component Signal Transduction Systems; TPS: Two-Partner System.

## Competing interests

The authors declare that they have no competing interests.

## Authors’ contributions

IP and MR contributed to manual annotation of the genome, analysed the data, drafted part of the manuscript and coordinated the annotation project. IP also performed SSH experiments. VB, AC, SM, BS (Segurens) performed sequencing of the genome. SC (Carrere) and JG performed automatic annotation of the genome and OrthoMCL analysis. RK contributed to manual annotation of the genome and drafted part of the manuscript. CM, VV and MA conceived the study and revised the manuscript. AD, M-A J, EL, SP, BS (Szurek) contributed to manual annotation of the genome and revised the manuscript. DWG conceived and supervised the SSH study. PR and SC (Cociancich) conceived the study, contributed to manual annotation of the genome and drafted part of the manuscript. All authors read and approved the final manuscript.

## Supplementary Material

Additional file 1 **List of proteins from *X. albilineans *strain GPE PC73 putatively T2SS-secreted based on SignalP 4.0 prediction [**http://www.cbs.dtu.dk/services/SignalP/**].** Click here for file

Additional file 2 **LPS gene cluster comparison between *X. albilineans *strain GPE PC73, *X. campestris *pv. *campestris *strain B100 and *X. campestris *pv. *vasculorum *strain NCPPB702.** Orthologs shared by at least two species are represented by arrows with identical colours. Specific genes for each species are shown by specific and neutral colour: grey for *X. campestris* pv. *campestris*, sky blue for *X. albilineans* and beige for *X. campestris* pv. *vasculorum*. HP = hypothetical protein. IS = insertion sequence. Click here for file

Additional file 3 **Chemotaxis gene cluster comparison between *Xanthomonas *species.** Orthologs shared by at least two species are represented by arrows with identical colours; the length of each arrow is proportional to the length in amino acids of each ORF. Specific proteins are shown by specific colours: grey for hypothetical protein, black for IS element or transposases, all other colours except pink for Methyl-accepting proteins and pink for other chemotaxis proteins. Click here for file

Additional file 4 **List of two-component signal transduction system identified in the genome of strain GPE PC73 of *X. albilineans *.** Two-component system regulatory proteins (I), two-component system sensor proteins (II) and two-component system sensor-response regulator hybrid proteins (III) are listed. Click here for file

Additional file 5 **List of transposases found in the chromosome of the strain GPE PC73 of *X. albilineans*.** The ORFs named “ISXal” are specific to *X. albilineans*. However, the recent publication of the complete genome sequence of *X. oryzae* pv. *oryzicola* revealed that XALc_2324, XALc_2365, XALc_2622 and XALc_2931, corresponding to ISXal5, ISXal7 and ISXal9 in *X. albilineans* strain GPE PC73, showed similarities with transposases found in the strain BLS256 of *X. oryzae* pv. *oryzicola.* Click here for file

Additional file 6 **CRISPR-1 and CRISPR-2 spacer distribution in *X. albilineans *strain GPE PC73.** Each box represents a CRISPR spacer, with the spacer positions numbered inside each box from the trailer end spacer to the leader end spacer. Pink boxes = CRISPR-1 spacers. Green boxes = CRISPR-2 spacers. Spacers showing nucleic acid identity with sequences of strain GPE PC73 of *X. albilineans* are listed in three separated tables according to the origin of these sequences (a same prophage region located between XALc_0170 to XALc_0242, phage and plasmid sequences and a housekeeping gene, respectively). Some spacers are 100% identical to corresponding sequences of strain GPE PC73 of *X. albilineans.* Percentage value is indicated in brackets only for spacers for which the percentage identity is less than 100%. Click here for file
