# Supplementary figures and images for: Genomic insights into strategies used by Xanthomonas albilineans with its reduced artillery to spread within sugarcane xylem vessels
Source: BMC Genomics. 2012 Nov 21;13:658. doi: 10.1186/1471-2164-13-658 (PMC3542200; doi:10.1186/1471-2164-13-658)

*X. campestris* pv. *campestris* str. B100

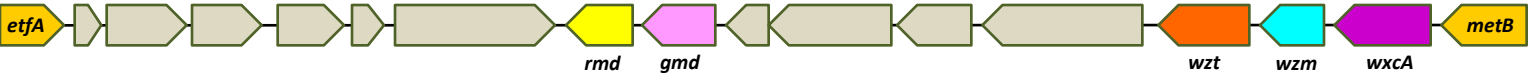

*X. albilineans* str. GPE PC73

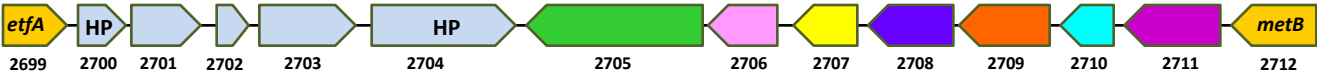

*X. campestris* pv. *vasculorum* str. NCPPB702

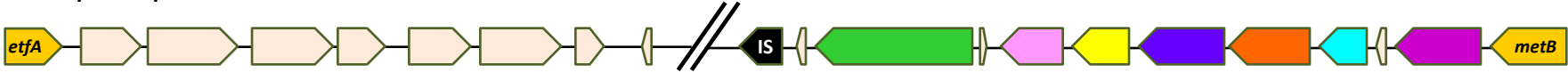

Supplement: Additional file 2 — LPS gene cluster comparison between X. albilineans strain GPE PC73, X. campestris pv. campestris strain B100 and X. campestris pv. vasculorum strain NCPPB702. Orthologs shared by at least two species are represented by arrows with identical colours. Specific genes for each species are shown by specific and neutral colour: grey for X. campestris pv. campestris, sky blue for X. albilineans and beige for X. campestris pv. vasculorum. HP = hypothetical protein. IS = insertion sequence. [file 1471-2164-13-658-S2.pdf]
