# Supplementary material for: Genomic insights into strategies used by Xanthomonas albilineans with its reduced artillery to spread within sugarcane xylem vessels
Source: BMC Genomics. 2012 Nov 21;13:658. doi: 10.1186/1471-2164-13-658 (PMC3542200; doi:10.1186/1471-2164-13-658)
Supplement: Additional file 3 — Chemotaxis gene cluster comparison between Xanthomonas species. Orthologs shared by at least two species are represented by arrows with identical colours; the length of each arrow is proportional to the length in amino acids of each ORF. Specific proteins are shown by specific colours: grey for hypothetical protein, black for IS element or transposases, all other colours except pink for Methyl-accepting proteins and pink for other chemotaxis proteins. [file 1471-2164-13-658-S3.pdf]

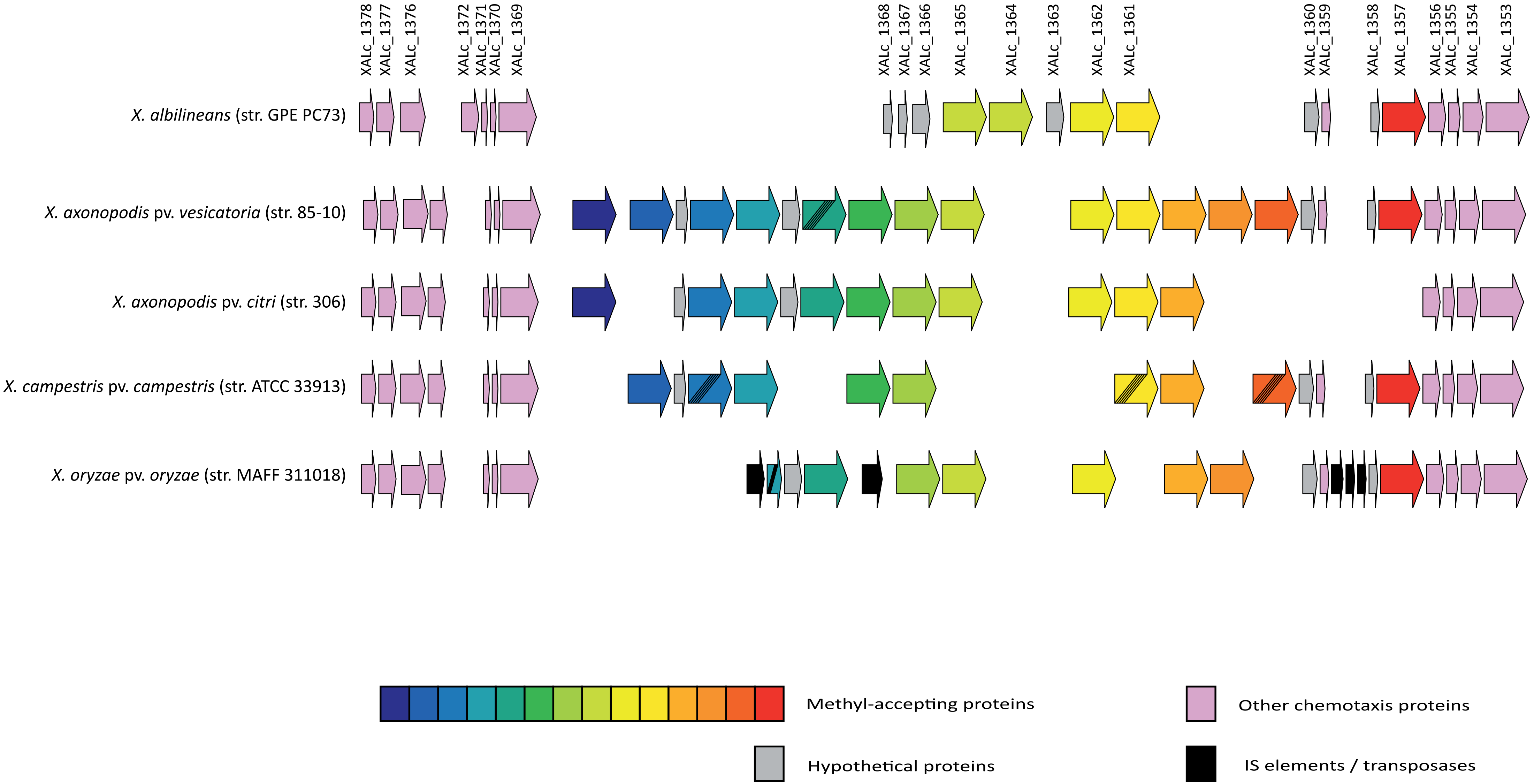
XALc_1378
XALc_1377
XALc_1376
XALc_1372
XALc_1371
XALc_1370
XALc_1369
XALc_1368
XALc_1367
XALc_1366
XALc_1365
XALc_1364
XALc_1363
XALc_1362
XALc_1361
XALc_1360
XALc_1359
XALc_1358
XALc_1357
XALc_1356
XALc_1355
XALc_1354
XALc_1353
X. albilineans (str. GPE PC73)
X. axonopodis pv. *vesicatoria* (str. 85-10)
X. axonopodis pv. *citri* (str. 306)
X. campestris pv. *campestris* (str. ATCC 33913)
X. oryzae pv. *oryzae* (str. MAFF 311018)
Methyl-accepting proteins
Other chemotaxis proteins
Hypothetical proteins
IS elements / transposases
