# Supplementary material for: Genomic insights into strategies used by Xanthomonas albilineans with its reduced artillery to spread within sugarcane xylem vessels
Source: BMC Genomics. 2012 Nov 21;13:658. doi: 10.1186/1471-2164-13-658 (PMC3542200; doi:10.1186/1471-2164-13-658)
Supplement: Additional file 6 — CRISPR-1 and CRISPR-2 spacer distribution in X. albilineans strain GPE PC73. Each box represents a CRISPR spacer, with the spacer positions numbered inside each box from the trailer end spacer to the leader end spacer. Pink boxes = CRISPR-1 spacers. Green boxes = CRISPR-2 spacers. Spacers showing nucleic acid identity with sequences of strain GPE PC73 of X. albilineans are listed in three separated tables according to the origin of these sequences (a same prophage region located between XALc_0170 to XALc_0242, phage and plasmid sequences and a housekeeping gene, respectively). Some spacers are 100% identical to corresponding sequences of strain GPE PC73 of X. albilineans. Percentage value is indicated in brackets only for spacers for which the percentage identity is less than 100%. [file 1471-2164-13-658-S6.pdf]

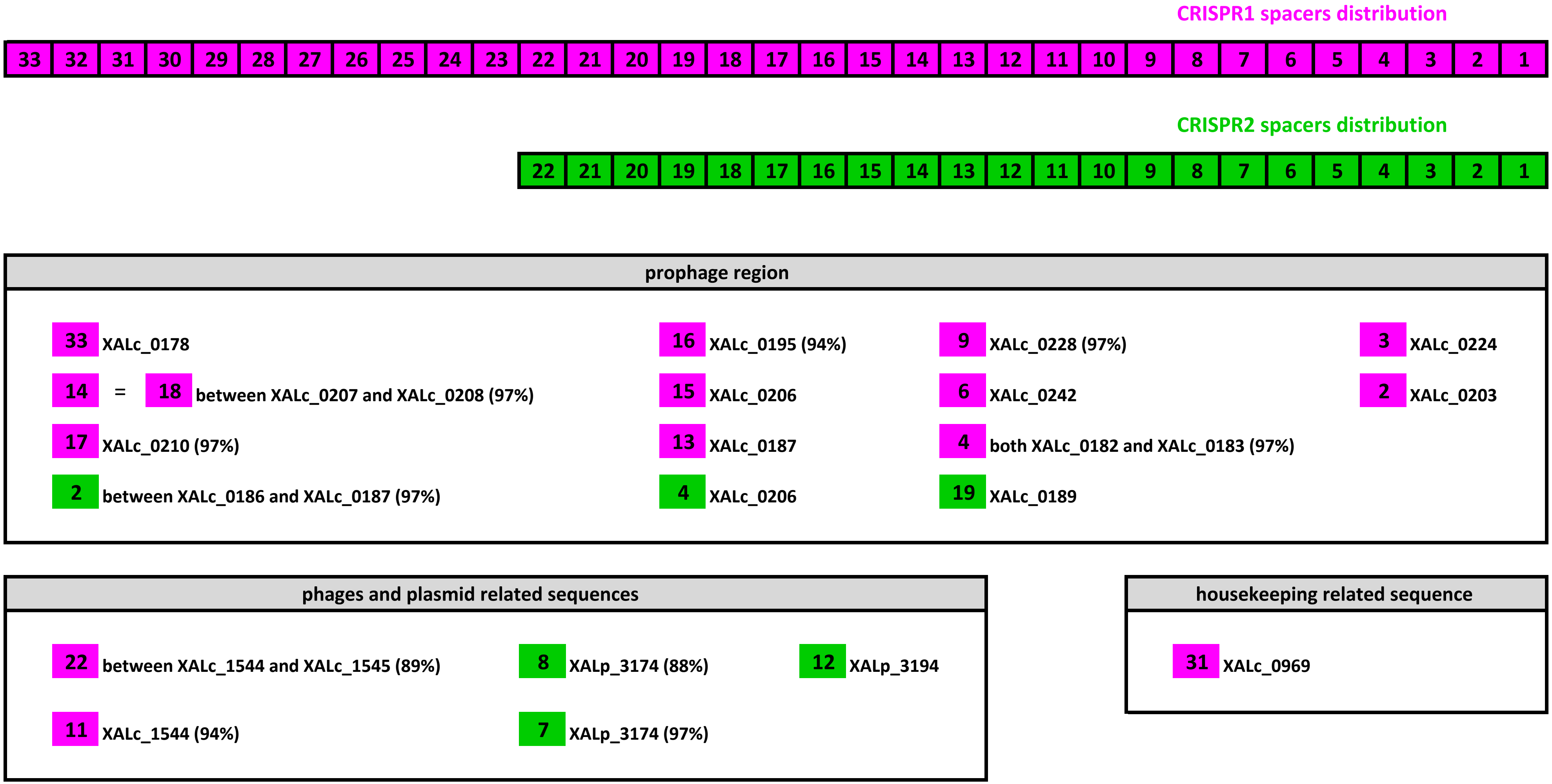

CRISPR1 spacers distribution
33 32 31 30 29 28 27 26 25 24 23 22 21 20 19 18 17 16 15 14 13 12 11 10 9 8 7 6 5 4 3 2 1
CRISPR2 spacers distribution
22 21 20 19 18 17 16 15 14 13 12 11 10 9 8 7 6 5 4 3 2 1
prophage region
33 XALc_0178
14 = 18 between XALc_0207 and XALc_0208 (97%)
17 XALc_0210 (97%)
2 between XALc_0186 and XALc_0187 (97%)
16 XALc_0195 (94%)
15 XALc_0206
13 XALc_0187
4 XALc_0206
9 XALc_0228 (97%)
6 XALc_0242
4 both XALc_0182 and XALc_0183 (97%)
19 XALc_0189
3 XALc_0224
2 XALc_0203
phages and plasmid related sequences
22 between XALc_1544 and XALc_1545 (89%)
11 XALc_1544 (94%)
8 XALp_3174 (88%)
7 XALp_3174 (97%)
12 XALp_3194
housekeeping related sequence
31 XALc_0969
